# Identification of moderate effect size genes in autism spectrum disorder through a novel gene pairing approach

**DOI:** 10.1038/s42003-026-10380-z

**Published:** 2026-05-30

**Authors:** Madison Caballero, F. Kyle Satterstrom, Silvia de Rubeis, Joseph D. Buxbaum, Behrang Mahjani

**Affiliations:** 1 Seaver Autism Center for Research and Treatment, Icahn School of Medicine at Mount Sinai, New York, NY USA; 2 The Mindich Child Health and Development Institute, Icahn School of Medicine at Mount Sinai, New York, NY USA; 3 Department of Psychiatry, Icahn School of Medicine at Mount Sinai, New York, NY USA; 4 Program in Medical and Population Genetics, Broad Institute of MIT and Harvard, Cambridge, MA USA; 5 Department of Genetics and Genomic Sciences, Icahn School of Medicine at Mount Sinai, New York, NY USA; 6 Analytic and Translational Genetics Unit, Department of Medicine, Massachusetts General Hospital, Boston, MA USA; 7 Friedman Brain Institute, Icahn School of Medicine at Mount Sinai, New York, NY USA; 8 Stanley Center for Psychiatric Research, Broad Institute of MIT and Harvard, Cambridge, MA USA; 9 Department of Neuroscience, Icahn School of Medicine at Mount Sinai, New York, NY USA; 10 Department of Artificial Intelligence and Human Health, Icahn School of Medicine at Mount Sinai, New York, NY USA; 11 Department of Molecular Medicine and Surgery, Karolinska Institutet, Stockholm, Sweden; 12 Department of Medical Epidemiology and Biostatistics, Karolinska Institutet, Stockholm, Sweden

**Keywords:** Genetics, Behavioural genetics

## Abstract

Autism Spectrum Disorder (ASD) arises from complex genetic and environmental factors, with inherited genetic variation playing a substantial role. This study introduces a novel approach to uncover moderate effect size (MES) risk genes in ASD, which individually do not meet the ASD liability threshold but contribute to risk when paired with another MES risk gene. Analyzing 10,795 families from the SPARK dataset, we identified 97 MES risk genes forming 50 significant gene pairs, demonstrating a substantial association with ASD when considered jointly, but not individually. Our method leverages familial inheritance patterns and statistical analyses, refined by comparisons against control cohorts, to elucidate these gene pairs’ contribution to ASD liability. Furthermore, expression profile analyses of these genes in brain tissues underscore their relevance to ASD pathology. This study underscores the complexity of ASD’s genetic landscape, suggesting that gene combinations, beyond high impact single-gene mutations, significantly contribute to the disorder’s etiology and heterogeneity. Our findings pave the way for new avenues in understanding ASD’s genetic underpinnings and developing targeted therapeutic strategies.

## Abstract (web summary)

Pairwise inheritance modelling uncovers moderate-effect autism spectrum disorder genes lacking single-gene association but jointly conferring risk, identifying 97 genes in 50 pairs and highlighting subthreshold genetic effects.

## Introduction

Autism Spectrum Disorder (ASD) is a complex neurodevelopmental disorder characterized by challenges in social interaction and communication, as well as restricted and repetitive behaviors. Extensive research has underscored the complex interplay of genetic and environmental factors contributing to its risk^1–6^. Environmental influences, including pregnancy complications, parental age, pollutants, and socioeconomic status, contribute to overall risk^7^. Nonetheless, there is consensus that genetic factors exert greater influence on ASD’s etiology, underscored by its substantial heritability exceeding 50%^2,8^. Large-scale genomic studies have revealed that ASD is a polygenic disorder influenced by a broad spectrum of genetic variants in the population rather than a single gene mutation^9^. These variants, ranging in effect sizes, collectively shape the likelihood and clinical manifestations of ASD. The threshold liability model, defined by an individual’s unique combination of genetic and environmental factors, has become the predominant way to understand and evaluate the complex interplay of influences contributing to ASD risk^10^. Thus, the likelihood of developing ASD is dependent on whether the summation of these factors crosses the threshold for an ASD phenotype.

Genetic factors contributing to ASD liability range in population frequency and effect size. Inherited common variants (those with a population frequency of at least 1%) explain approximately 50% of ASD liability in the population^1,2,6^. Though common variants play a substantial role when considered together, an individual common variant provides little contribution to individual liability and often offers little biological insight. The pressure of negative selection forces variants that individually confer substantial heritability for ASD to be rare in the population; thus, studies of rare variants ranging from single nucleotide variants to large structural changes have been undertaken to help identify risk genes and developmental pathways. In recent years, research efforts have focused on identifying de novo mutations in particular. De novo loss-of-function variants are rare in the general population, particularly in genes which are haploinsufficient or which exhibit evolutionary constraint. Genes in which such variants are overrepresented in individuals with ASD can be identified as ASD risk genes, and the variants themselves can confer extreme phenotypes that provide meaningful biological insight^3,4^. However, de novo mutations only account for approximately 5% of ASD cases^2^, and their sporadic origin renders them less representative of ASD’s overall heritability.

While 1,100 genes are estimated to contribute to ASD risk^11^, only a few hundred have been confidently identified^3,4,6,12^, mostly by considering aggregate variants across a gene and leveraging advanced statistical models such as the Transmission and De Novo Association (TADA) test^13^. TADA increases statistical power by combining de novo mutations and case-control inherited variants while incorporating per-gene mutation rates and constraint scores^14,15^. Driven by variants of large effect, TADA has significantly improved the identification of genes associated with ASD^4^. However, this approach loses power when considering genes where deleterious variants are inherited and confer smaller effect sizes, as these variants are more frequently found in non-ASD controls. As a result, there is a gap in our understanding of genes where variants make moderate, transmittable contributions to ASD liability.

**Fig. 1:**
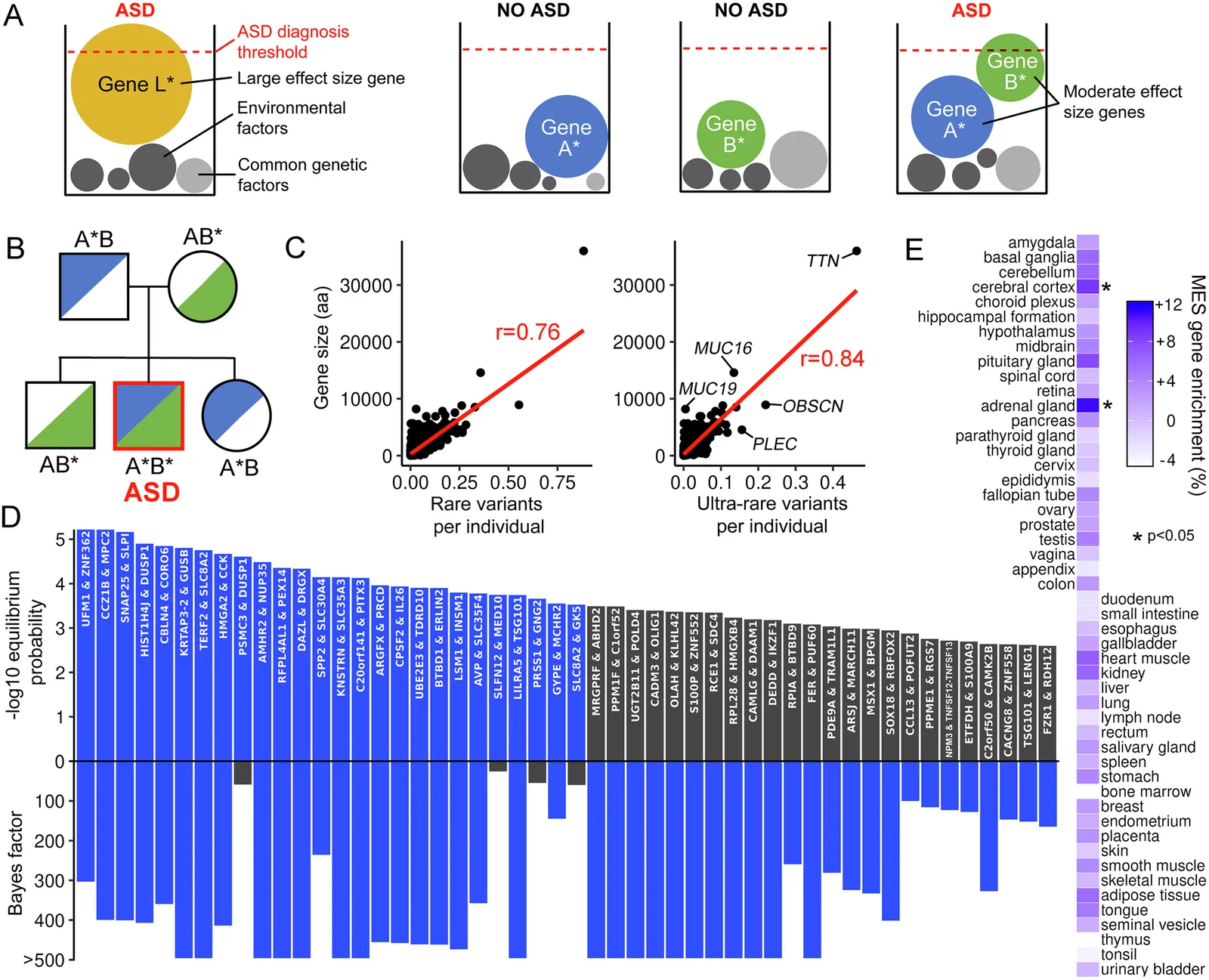
Discovery and enrichment of MES risk genes. **A** Depiction of gene effect size in the context of cumulative risk factors contributing to individual ASD liability. Adapted from Huang et al. (2024)^51^. Deleterious variants in large effect genes exhibit near-complete penetrance and can independently exceed the diagnostic threshold. In contrast, individual MES risk genes confer modest effects insufficient on their own to reach liability; however, we hypothesize ASD may arise when deleterious variants in two or more distinct MES genes co-occur. **B** Example inheritance pattern illustrating ultra-rare deleterious variants in two genes within the parents (depicted in blue or green) that are exclusively jointly inherited by offspring with ASD. This exemplifies a potential genetic mechanism contributing to ASD susceptibility. **C** Mean number of rare (<1%) or ultra-rare (<0.1%) deleterious variants in SPARK parents versus gene size. The red line represents linear regression. Without *TTN*, correlation is 0.71 and 0.80, respectively. **D** Equilibrium probability and Bayes factors for the predicted MES risk gene pairs in ASD. Bars in blue denote an equilibrium probability <3.03 × 10^-4^ or a Bayes factor >100. **E** Enrichment of predicted MES risk genes per tissue relative to all protein-coding genes.

Here we propose and implement a method to identify moderate effect size (MES) risk genes in ASD. We define MES risk genes as genes that individually do not surpass the ASD liability threshold when disrupted and therefore have no significant association when tested in isolation (Fig. 1A). This operational definition accommodates differences in gene size and variant frequency that affect discovery power. In practice, this corresponds to inherited effects that are typically below single-gene exome-wide detectability at current sample sizes. We reason that co-inheritance of deleterious variants in two MES risk genes, together with common genetic and environmental background, can elevate liability above the ASD threshold. Based on this model, we built a pipeline to identify gene pairs enriched in ASD cases and applied it to empirical data to test whether it yields statistically meaningful results under null expectations. Importantly, our pair-based strategy does not imply functional interaction or epistasis. Rather, the pipeline is indifferent to the relationship between the genes, and we later evaluate whether identified pairs are more consistent with additive or dependent effects.

The process of testing gene combinations poses a statistical challenge, particularly given the vast number of potential gene pairs. With approximately 20,000 protein-coding genes, the number of two-gene combinations is approximately 200 million, necessitating an extremely stringent significance threshold. To overcome this challenge, we first selected candidate pairs based on familial inheritance patterns of ultra-rare deleterious variants, narrowing the focus to a manageable subset of potential risk genes that relies on empirical expectations of deleterious variant frequency and transmission probability. Subsequent statistical analysis on this subset aimed to detect gene pairs more prevalent in individuals with ASD compared to a control cohort with over 200,000 individuals. We find evidence that pairs are predominantly independent and do not necessarily form dependent or specific pairings. After establishing statistical significance and validating findings, we then characterized 97 predicted MES risk genes to further support their roles in ASD etiology and heterogeneity.

## Results

### Identification of candidate MES risk genes inherited as pairs

We leveraged familial inheritance patterns of ultra-rare deleterious variants in search for candidate MES risk genes. Our strategy identified instances where variants in two distinct genes were inherited in offspring with ASD, with non-ASD offspring potentially inheriting either variant, but not both. For this, we used the SPARK^16^ iWES v1 dataset (“SPARK v1”), a cohort of 70,487 individuals of which 34,164 have a diagnosis of ASD. We initially analyzed 10,795 families within SPARK, each with both parents and at least one ASD offspring genotyped with whole exome sequencing data (WES; see **Methods**).

**Fig. 2:**
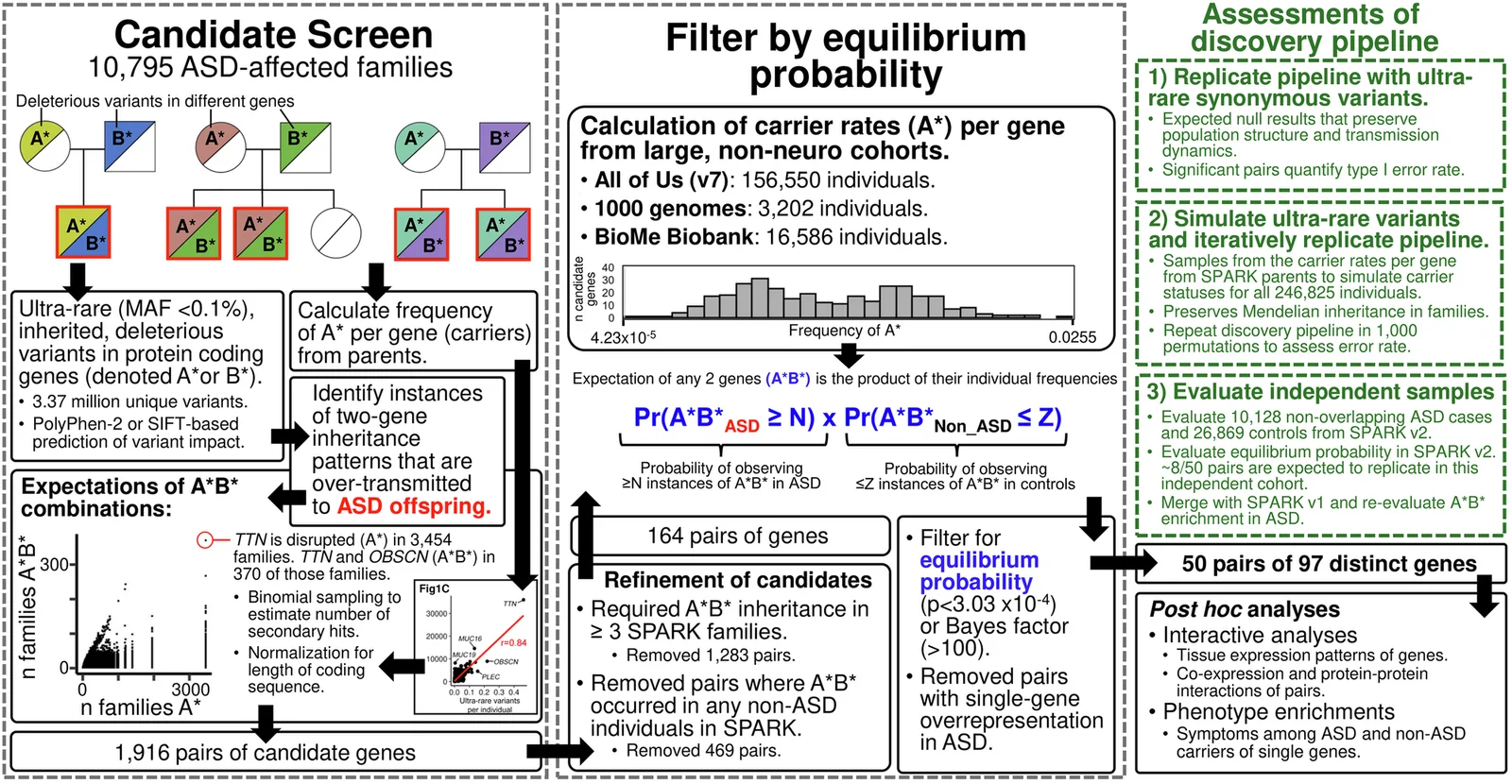
Diagram of steps involved in MES risk gene discovery and statistical assessment of false positive rate.

The first and second steps of our candidate screen identified familial structures wherein ultra-rare (frequency <0.1%) deleterious variants in two genes were inherited by all offspring with ASD (Figs. 2 and  1B). In this context, the term ‘A*‘ designates an ultra-rare deleterious genetic alteration in a given gene present in one parent, with the potential for transmission to the offspring. The second step searched for mirrored inheritance patterns involving an ultra-rare deleterious variant in a different gene, denoted as ‘B*’. In such instances, ‘B*‘ originates from the parent who did not transmit ‘A*’, thereby maintaining genetic independence. We then identified instances where all ASD offspring that share the same parents exhibited ‘A*B*‘ inheritance, while none of their non-ASD siblings carried both alleles.

In the third step of our candidate screen, to identify pairs for further analysis, we assessed the likelihood of observing the incidence of mirrored ‘B*‘ inheritance within families displaying ‘A*‘ inheritance. This entailed calculating the likelihood of ‘B*‘ variants manifesting in a second gene, contingent upon our knowledge of the prevalence of such variants in the population. For instance, let us consider ‘GeneA’ which features ‘A*‘ inheritance in seven families, and ‘GeneB,’ with ‘B*‘ inheritance in 20 families. Among these, four families exhibited mirrored ‘A*B*‘ inheritance pattern. Utilizing the binomial distribution and adjusting by the frequency of ultra-rare deleterious variants per gene in the SPARK parent population, we determined (1) the expected frequency of observing this four-in-seven and, conversely, four-in-twenty arrangement, and (2) the relative likelihood that the genes observed were ‘GeneA’ and ‘GeneB’ (see **Methods**). This latter step is essential as genes with longer coding sequences generally have a greater frequency of ultra-rare deleterious variants (Fig. 1C). To illustrate, ‘A*‘ inheritance patterns within the large gene *TTN* were observed in 32.0% of all SPARK families. Consequently, the occurrence of a mirrored ‘A*B*‘ inheritance pattern with *TTN* is considerably more anticipated than with *SNAP25*, a small gene where ‘A*‘ inheritance patterns were present in only 0.048% of the SPARK families. We retained 1,916 pairs of genes where the normalized expected frequency was less than one in both ‘A*‘ and ‘B*‘ configurations (Data S1).

In the fourth step, we stipulated that a minimum of three families must display the ‘A*B*‘ inheritance pattern for each candidate pair, ensuring robust statistical analyses by reducing bias from sparse observations. We further required that there were no occurrences of the ‘A*B*‘ pattern among the remaining non-ASD individuals within the broader SPARK cohort, which comprised 36,323 first-degree relatives of someone with ASD. This filtering process resulted in a final count of 164 candidate pairs from 316 unique protein coding genes (Data S2).

Of note, we tested additional variant classes and frequencies, including missense variants and PTVs of rare (<1%) frequency and analyses restricted to only PTVs (see **Methods**). We selected PTVs and missense variants of ultra-rare or rarer frequency for the following analyses as it produced the greatest number of candidate pairs after SPARK family filtering.

### Statistical assessment of MES risk genes and incorporating non-neuro cohorts

Though SPARK includes individuals without ASD, all are within families where at least one individual has ASD. This makes these individuals an inaccurate representation of the general population regarding genetic risk. Therefore, we included individuals without known mental, behavioral, or neurodevelopmental disorders. This included 3,202 individuals from the 1000 genomes project (1kGP)^17,18^, 156,550 individuals from the All of Us (v7) consortium, and 16,586 individuals from the BioMe Biobank. As with SPARK, we identified ultra-rare deleterious variants in the 316 candidate MES risk genes. To ensure comparability across control cohorts, we examined the frequency of ultra-rare deleterious variants in the candidate MES risk genes and found robust correlations and no population structure confounding (Figure S1; see Methods).

Among the 212,661 individuals without ASD across all cohorts, ‘A*B*’ was absent in 26 out of the 164 candidate pairs and occurred in only two or fewer individuals in 83 candidate pairs. Across all candidate pairs, the incidence of ‘A*B*’ was consistently higher among individuals with ASD than their non-ASD counterparts. Notably, in 71 pairs, the frequency of ‘A*B*’ was more than 10 times greater in individuals with ASD. Together, this illustrates a pronounced disequilibrium between individuals with ASD and those without ASD.

Our above search (Candidate screen in Fig. 2) aimed to identify potential MES risk genes for in-depth analysis, mitigating the influence of large genes with a high frequency of ultra-rare deleterious variants. In the fifth and final step, our objective was to assess the skew in MES risk gene pairs in relation to ASD. We hypothesized that the concurrent inheritance of ultra-rare deleterious variants in two paired MES risk genes was strongly associated with ASD development. We anticipated that the prevalence of ‘A*B*‘ in the population would adhere to an expected equilibrium, being the product of ‘A*‘ and ‘B*‘ frequencies in the population. Their significant enrichment in individuals with ASD is presumably the result of their involvement in ASD etiology. In contrast, our null hypothesis is that the overrepresentation of ‘A*B*‘ in individuals with ASD, contingent upon the individual frequencies of ‘A*‘ and ‘B*‘ in the population, occurred by chance. Furthermore, when examining the frequency of ‘A’ or ‘B’ in isolation, we required that neither gene be individually associated with ASD at p < 0.05. This ensures that the observed association arises from the co-inheritance of both variants rather than a strong effect from one gene alone. Therefore, with respect to ASD, we required only ‘A*B*’ to be in strong disequilibrium.

To illustrate, consider ultra-rare deleterious variants in the genes *SNAP25* (as ‘A*‘) and *SLPI* (as ‘B*‘), which are present in 0.056% and 0.196% of individuals, respectively. Notably, these frequencies do not show significant differences when queried in individuals with or without ASD (0.053% versus 0.057% for *SNAP25* and 0.220% versus 0.192% for *SLPI*, respectively). At equilibrium, ‘A*B*‘ should occur at a frequency determined by the product of their individual frequencies. For *SNAP25* and *SLPI*, ‘A*B*’ occurs in three of 34,164 individuals with ASD and in none of the 212,661 individuals without ASD. Using the cumulative density function of the binomial distribution, we calculated the probability of observing at least three individuals with ASD having ‘A*B*‘ and none without ASD to be 6.88x10^-6^. In addition, we calculated the Bayes factor for each pair. As described above, our null hypothesis posits that the increased occurrence of ‘A*B*‘ in individuals with ASD is a random event occurring at the equilibrium probability observed above. Our alternate hypothesis is that ‘A*B*’ is enriched among individuals with ASD precisely because it results in an ASD phenotype. Using the example of *SNAP25* and *SLPI*, the Bayes factor was 400, strongly supporting the alternate hypothesis. Interestingly, one of the largest Bayes factors was 5,807 for the genes *PITX3*, encoding a transcription factor with a known role in the development and maintenance of midbrain dopaminergic neurons^19^, and *C20orf141*, a gene of unknown function.

We retained 50 candidate gene pairs (97 unique genes) that met significance thresholds of equilibrium probability < 3.03 × 10^-4^ (corrected for 164 tests) or Bayes factor >100 (Fig. 1D; Data S3). Across these pairs, 183 individuals with ASD carried at least one A*B* configuration, comprising ~0.5% of ASD cases analyzed. Notably, while these genes show significant pairwise associations with ASD, none reached significance when tested individually. We refer to them hereafter as ‘predicted MES risk genes.’

According to the recent and most comprehensive screens for ASD genes^4^, the mean ASD FDR of the predicted MES risk genes was 0.804, demonstrating that single MES risk genes by themselves are not generally associated with ASD. Only one gene, *DEDD*, had an ASD FDR < 0.05. Six genes (*PUF60, SNAP25, PSMC3, CAMK2B*, and *HIST1H4J*) were associated with neurodevelopmental disorders (FDR < 0.05) though not for ASD. Similarly, the 97 MES risk genes were also not associated with ASD in other major studies^3,11,12,20^ and are therefore not considered consensus ASD-associated genes.

The median probability of being loss-of-function intolerant (pLI) score of the predicted MES risk genes was 0.029 and only 15 genes were >0.9, the common threshold for haploinsufficiency. MES risk genes inhabited more moderate pLI deciles than large effect size genes characterized in the most recent comprehensive assessment of ASD genes^4^ (Figure S2). This was also the same result for loss-of-function observed over expected upper bound fraction (LOEUF) scores (Figure S2). This suggests the predicted MES risk genes are relatively less constrained and more haplosufficient. This is again consistent with our model of MES risk genes where each gene alone is not evidently associated with ASD. Importantly, bialleic deleterious variants are not present nor assessed in this study as they may yield separate pathological phenotype. Nonetheless, we present 97 predicted MES risk genes that when disrupted in concert with a second MES risk gene are strongly associated with ASD.

### Controlling false positives in candidate gene pair analysis

To mitigate the challenge of a highly stringent multiple testing correction threshold inherent in comparing every protein-coding gene, we streamlined our analysis to focus on candidate gene pairs. This approach, while efficient, raises concerns of bias due to selective inference, as it prioritizes pairs of genes with a pre-established likelihood of association. To address potential bias, we implemented three validation strategies: (1) Conducting a replication study using synonymous variants as a neutral benchmark; (2) Evaluating pairs in an independent ASD cohort; and (3) Simulating associations to establish an accurate null distribution.

Our initial validation utilized ultra-rare synonymous variants, which, due to their non-functional impact, were anticipated to yield fewer or less significant associations with ASD. This analysis was conducted identically to that for deleterious variants. We found a similarly strong correlation between the quantity of ultra-rare synonymous variants and CDS length (Pearson r: 0.83), as well as between the counts of ultra-rare synonymous and deleterious variants (r: 0.82; Figure S3A, B). Moreover, the variability in the frequency of ultra-rare synonymous variants across different cohorts paralleled that observed for missense variants (Figure S3C), affirming that synonymous variants replicate our analyses under the same conditions. Utilizing ultra-rare synonymous variants, we identified merely five gene pairs (10 unique genes; Data S1D), in contrast to the 50 pairs identified with deleterious variants. Together, this suggests a potential false-positive rate of approximately 10%.

Further validation involved a new cohort of 36,997 non-overlapping samples from SPARK (“SPARK v2”), including 10,128 ASD individuals. Simulations indicate an average of 8.6 pairs (standard deviation of ±2.7 pairs) to have at least one A*B* in 10,128 independent ASD cases (**Methods**). In line with these expectations, only eight of the original 50 gene pairs could be validated as A*B* was not observed in ASD for the other 42 pairs. Nonetheless, among the eight re-observed, seven pairs met the criteria of an equilibrium probability <0.05 or a Bayes Factor >10, adjusted for the smaller independent dataset (Figure S4A). None of the five pairs identified with ultra-rare synonymous variants were re-observed. In addition, by combining data from both the SPARK v1 and v2 cohorts, we found 38 of the original 50 pairs maintained significance (equilibrium probability <3.03 × 10^-4^ or a Bayes factor >100; Data S3; Figure S4B). By contrast, only two synonymous variant pairs retained an association post-combination, none surpassing an equilibrium probability of 1.2 x 10^-4^, a stark contrast to the maximum significance of 5.9 x 10^-8^ observed in the primary analysis with ultra-rare deleterious variants (Data S4).

Finally, to assess the significance of the 50 observed MES pairs, we simulated ultra-rare variants in 1,000 permutations and reran our MES discovery pipeline (Fig. 2). This simulation samples from the observed distributions of ultra-rare deleterious variants, creating a more accurate null distribution of pair associations. Using the per-gene carrier rates from non-ASD SPARK parents, we simulated carrier statuses for all 246,825 individuals assessed in this study, conserving familial relationships and Mendelian inheritance patterns (**Methods**). Though we did not find significantly fewer pairs in the simulations, the equilibrium probability and Bayes factor of ASD association for the true 50 MES pairs was lower and higher on average, respectively. Across all 1000 simulations, we found the association of true MES pairs to ASD was significantly higher (p < 0.05, Wilcoxon rank sum test) (Figure S4C). Together, these results demonstrate that our pipeline identifies MES risk gene pairs that are more associated with ASD than would be expected under an apt null distribution.

To summarize, our methodology efficiently identifies MES risk genes while maintaining a low false-positive rate and higher significance than expected under a simulated null distribution. Of the initial 50 gene pairs, 38 (74 unique genes) were corroborated by integrating the SPARK v2 dataset, with seven out of eight subjected to independent validation using SPARK v2. The limited replication observed in an independent cohort is consistent with statistical expectations given the rarity of these variants and the smaller sample size. This does not indicate a lack of association but rather reflects the challenge of validating rare variant effects in datasets of limited size. Subsequent analyses will delve into the 97 unique genes within these pairs, focusing on their expression patterns and phenotypes among carriers as further evidence of their involvement in ASD.

### Enrichment of MES risk gene expression in the brain

To further our identification of MES risk genes implicated in ASD, we conducted an analysis of their tissue expression profiles. While ASD etiology encompasses factors beyond neural influences, it is imperative that genes contributing to ASD development exhibit overrepresented expression in the brain. Accordingly, we hypothesized that our predicted MES risk genes would exhibit an enrichment for expression in brain tissues.

Utilizing consensus normalized expression values (nTPM) from the Human Protein Atlas (v23)^21^, and encompassing 50 tissues, we observed that 77.0% of the 20,151 protein coding genes demonstrated detectable expression (nTPM > 1) in at least one brain region, with 70.5% exhibiting expression in the cerebral cortex. To assess the statistical significance of these findings, we performed random gene sampling and quantified per-tissue expression, revealing overrepresentation of predicted MES risk genes in the cerebral cortex (p = 0.022) and the adrenal glands (*p* = 0.009) (Fig. 1E; Figure S5A). The predicted MES risk genes did not exhibit significant underrepresentation in any tissues. Of note, we did not correct for multiple-testing correction due to expression correlation across other brain regions, indicating similar MES gene overrepresentation (e.g., midbrain *p* = 0.053). While cerebral cortex enrichment was expected, adrenal gland expression may implicate stress-response pathways. A recent meta-analysis confirms elevated peripheral cortisol in ASD^22^, suggesting possible neuroendocrine contributions to ASD liability.

Among the 10 false-positive genes identified with synonymous variants, we did not observe over or underrepresentation in any tissue. Among the 74 unique genes that maintained significance with the addition of SPARK v2, we observed the same cerebral cortex and adrenal gland enrichment. Furthermore, we did not observe that the predicted MES risk genes were expressed at a significantly higher level than other cerebral cortex or adrenal expressed genes. Nonetheless, the observed enrichment of MES risk gene expression in the cerebral cortex aligns with our initial predictions.

To conduct a more in-depth investigation, we queried gene expression within 193 subregions of the brain (Figure S5B). Notably, we identified the most pronounced enrichment of the 97 predicted MES risk genes in the ventral tegmental area and central amygdala, although the statistical significance fell just below the conventional threshold (*p* = 0.08 and *p* = 0.07, respectively). Again, the same pattern was observed with the 74 unique genes that maintained significance with SPARK v2. It is noteworthy that these brain regions exhibited the highest proportion of MES risk genes with detectable expression (83.5% for both regions), surpassing the expression levels observed in other brain regions or any alternative tissues that we analyzed. The 97 predicted MES risk genes are not canonically associated with ASD because they do not show strong associations when tested alone. Yet, their expression is most pronounced in a region of the brain linked to psychiatric disorders including ASD^23,24^. Together, we showed the predicted MES risk genes are characterized by expression in neural tissues, suggesting their disruptions may result in psychiatric phenotypes.

### Phenotypes of non-ASD carriers of MES risk genes

Identifying genes in pairs suggests two types of associations: dependent and independent pairings. Dependent pairing, involving mechanisms like epistatic or gene-gene interactions, would likely result in exclusive pairings, with evidence of interactions such as co-expression and no phenotypic differences in carriers of only one gene. In contrast, independent pairing, where each gene contributes separately to the risk, would allow for more permissive gene pairings and potentially detectable phenotypes in individuals with a mutation in just one gene. The following sections will evaluate these competing models.

Independent MES risk genes should be capable of appearing in multiple pairing configurations. Conversely, the dependent model predicts exclusive, specific pairs. In our analysis of 50 pairs, we identified three genes (*DUSP1*, *SLC8A2*, and *TSG101*) appearing in multiple configurations with different partners. Through 100,000 sampling iterations with replacement, the likelihood of observing three or more duplicated genes from a pool of all protein coding genes was significantly higher than expected by chance (*p* = 0.0032). Consistent with this model, combinations of any two or more MES genes were enriched in ASD individuals in SPARK v1 and v2, and this enrichment remained after removing individuals carrying any of the 50 specific MES pair conformations (Data S5). We replicated this observation independently in both the Simons Simplex Collection^25^ (9,188 individuals; 2,415 with ASD) and the largest MSSNG sub-cohort (7,289 individuals; 3,313 with ASD), again finding individuals carrying two or more MES genes in any configuration were enriched among ASD cases relative to their unaffected family members (Data S5). Together, this supports the hypothesis that at least some MES risk genes are independent and do not form exclusive pairs.

In the scenario of independent contributions, the disruption of a single MES risk gene may manifest mild ASD-like phenotypes, while the disruption of two MES risk genes could result in an additive or synergistic effect that surpasses the ASD liability threshold. In contrast, within the context of dependent GxG interactions, the disruption of a single MES risk gene yields no discernible phenotype. To observe if any predicted MES pairs show independent contributions, we queried the phenotypes and questionnaire responses of non-ASD individuals within the SPARK v1 cohort. Per our MES risk gene discovery methodology, non-ASD individuals may have an ultra-rare deleterious variant in either gene (herein simply referred to as ‘carriers’) of an MES risk gene pair – but not both.

We first examined Social Communication Questionnaire (SCQ) responses, consisting of 40 yes-or-no items used for ASD screening, available for 3,831 non-ASD offspring in our study. Other ASD phenotyping evaluations were not available for non-ASD individuals. We focused on 68 out of the 97 predicted MES risk genes that exhibited at least five non-ASD carriers with SCQ responses. We found 31 out of the 68 tested genes exhibited a pronounced skew (χ^2^ *p* < 0.05) for at least one SCQ question (Figure S6). For 15 genes, more than one question exhibited significantly increased ASD-like responses, with a maximum of six skewed questions per gene. To assess the significance of these results, given the rarity and variable carrier numbers per gene, we shuffled the carrier statuses among non-ASD respondents across 1,000 iterations, maintaining consistent carrier numbers per gene. We then analyzed the number of ASD-skewed (χ^2^ *p* < 0.05) SCQ questions per gene using the cumulative density of the normal distribution (**Methods**). Six genes showed significantly (*p* < 0.05) more ASD-skewed responses than the shuffling permutations (Figure S6). For instance, non-ASD carriers of *SLC30A4* exhibited six questions with increased ASD-like responses, a significantly higher number (*p* = 6.3 x 10^-8^) than observed in the permutations.

In addition to examining individual SCQ questions, we assessed the total SCQ score. No discernible elevation in total scores was observed among carriers of any MES risk genes. The most substantial mean score difference between non-ASD carriers and non-carriers was less than two points. Nonetheless, our study demonstrated that non-ASD carriers of MES risk genes may exhibit significant differences in responses to specific SCQ questions, but not in overall scores.

Though ASD-related phenotype measurements were less abundant for non-ASD individuals, we were able to evaluate the presence of other psychiatric or developmental conditions. Across 28,883 non-ASD individuals and 29 questions on the presence of developmental or psychiatric conditions (e.g., schizophrenia, obsessive-compulsive disorder, sleep disorders, motor delays), we found 47 of 97 tested MES risk genes exhibited a skew (χ^2^ *p* < 0.05) for at least one condition (Figure S7). In 83.6% of cases, carriers of the MES risk gene showed increased incidence of at least one condition. For example, 15.5% of non-ASD carriers of ultra-rare deleterious variants in *PITX3* reported obsessive-compulsive disorder (OCD) compared to 4.3% of non-carriers (*p* = 0.004), in line with the critical role of *PITX3* in midbrain dopaminergic neurons^19^ and the evidence implicating circuits involving these neurons in OCD-like behaviors in animal models^26^. Of note, the minority of cases where MES risk gene carriers had significantly lower incidence occurred exclusively for conditions that were relatively common in the SPARK cohort such as depression and social anxiety. Using the same shuffling approach previously applied to SCQ questions, we evaluated the significance of skewed condition incidence, both in terms of the number and the directionality. Our analysis identified five genes with a significant (*p* < 0.05) increase in the number of conditions with elevated incidence in carriers and ten genes with a significant decrease in incidence. For instance, the increased incidence of three neurodevelopmental disorders—ADHD, oppositional defiance disorder, and learning disabilities—in non-ASD carriers of *TDRD10* was significantly elevated (*p* = 0.008).

### Phenotypes of ASD carriers of MES risk genes

The heightened prevalence of psychiatric conditions in non-ASD carriers of individual MES risk genes suggests a potential influence on the diverse presentation of ASD. It is conceivable that MES risk genes contribute to both the overall liability of ASD and the liability of comorbid symptoms associated with ASD. In the SPARK v1 cohort, 99.95% of individuals with ASD exhibit at least one of the 29 comorbid psychiatric or developmental disorders assessed. Notably, language delays are the most common, affecting 51% of individuals, followed by attention-deficit/hyperactivity disorder (ADHD) at 38%.

**Fig. 3:**
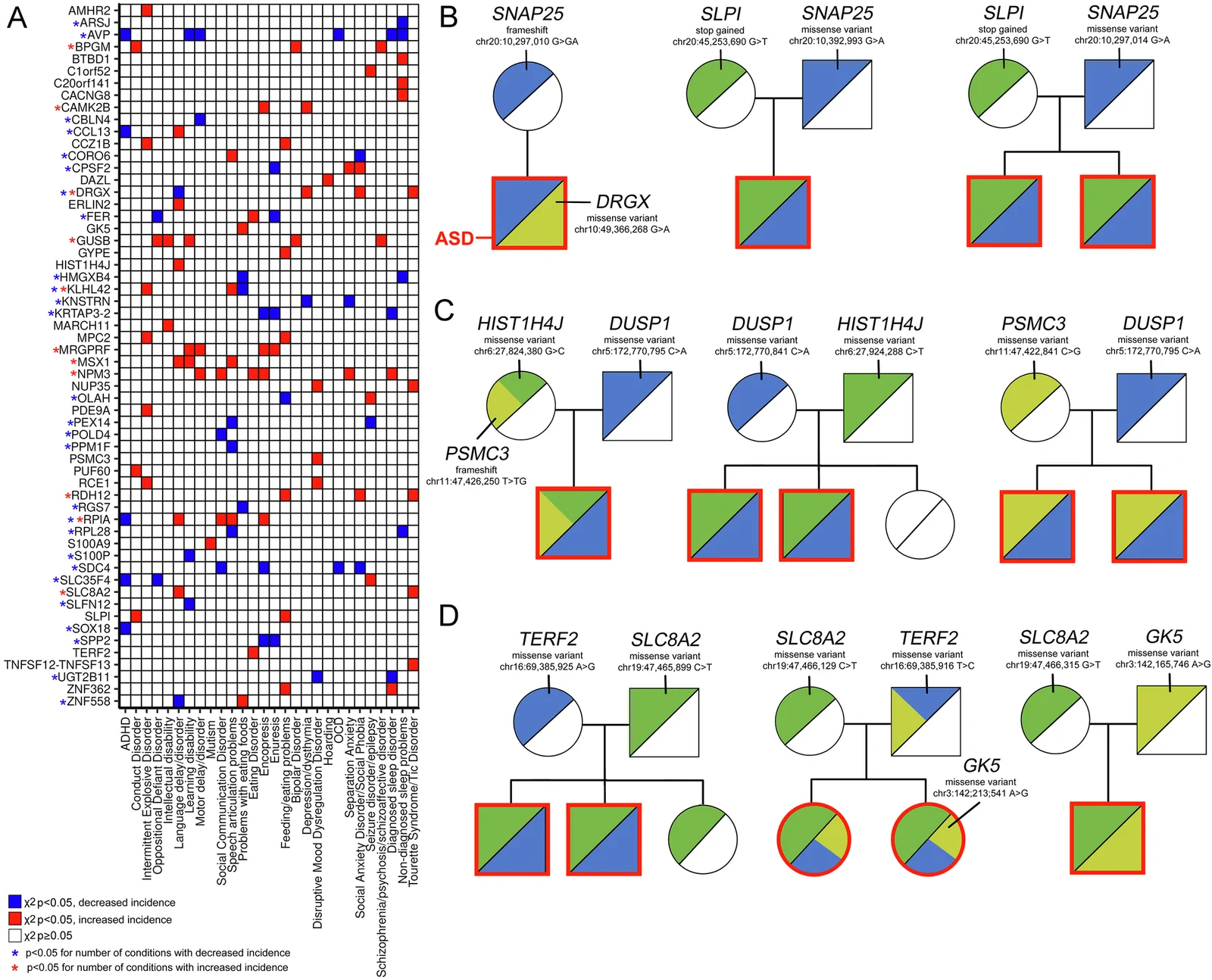
Phenotypes and familial inheritance of MES risk genes. **A** Differences in comorbidity frequency of carriers of MES risk genes with ASD. Genes with χ^2^ *p* < 0.05 differences in ASD carrier versus non-carrier condition frequencies are highlighted. Only genes with at least one instance of comorbidity bias are shown. Genes where the number of skewed responses increasing or decreasing incidence is significant (*p* < 0.05) compared to shufflings are noted with an asterisk. **B** Example families from SPARK v1 that carry mutations in *SNAP25*. **C** As in panel **B** for *DUSP1*. **D** As in **B** for *SLC8A2*.

Among 58 of the 97 MES risk genes analyzed, carriers with ASD displayed skewed frequencies (χ^2^ *p* < 0.05) of comorbid disorders compared to ASD individuals without any MES risk genes (Fig. 3A). For 31 MES risk genes, comorbid disorders were more prevalent in ASD carriers. For instance, individuals with ASD carrying an ultra-rare deleterious variant in the gene *NPM3* demonstrated a higher incidence of motor delay (χ^2^ *p* = 0.002), diagnosed sleep disorders (χ^2^ p = 0.007), separation anxiety (χ^2^ *p* = 0.02), social communication disorder (χ^2^ p = 0.02), eating disorders (χ^2^ *p* = 0.02), and encopresis (χ^2^ p = 0.03). Importantly, carriers of *NPM3* among non-ASD individuals also exhibited a significantly higher incidence of learning disabilities (χ^2^ p = 0.01) and separation anxiety (χ^2^ p = 0.04). In total, among the 31 MES risk genes associated with increased comorbidity frequency in individuals with ASD, 14 were also linked to increased comorbidity frequency in non-ASD individuals, though not always for the same comorbidity. Using the above-described approach of shuffling carrier statuses among individuals with ASD, we found 11 MES risk genes with a significant (*p* < 0.05) increase in the number of conditions with elevated incidence in carriers (Fig. 3A). For example, among ASD *NPM3* carriers, the presence of at least six conditions with increased incidence was highly significant (*p* = 3.7 x 10^-11^).

Interestingly, for 17 MES risk genes, individuals with ASD exclusively exhibited lower incidence of comorbidities and all were significant compared to random shufflings (Fig. 3A). Carriers of ultra-rare deleterious variants in *AVP*, a gene that produces the neuropeptide hormone arginine vasopressin, demonstrated a reduced incidence of ADHD (χ^2^ *p* = 5x10^-4^), sleep disorders (χ^2^ *p* = 0.004), OCD (χ^2^ *p* = 0.03), motor delays (χ^2^ *p* = 0.03), and learning disabilities (χ^2^ *p* = 0.048). Compared to the random shuffling, observing any six or more conditions with decreased incidence among *AVP* ASD carriers was immensely significant (*p* < 1x10^-100^). Notably, in non-ASD individuals, carriers of *AVP* exhibited a decreased incidence of depression (χ^2^ *p* = 0.02) and a noteworthy, albeit not statistically significant, decrease in ADHD and sleep disorders. As *AVP* is anxiogenic and promotes the stress response^27^, we tangentially hypothesize its disruption could reduce these effects. Its pronounced association with decreased comorbid psychiatric and neurodevelopmental disorders in ASD suggests this gene may be associated with milder phenotypes.

In addition, a further 10 MES risk genes showed increased frequencies for one comorbidity while decreased frequency for another. Carriers of *DRGX*, a gene involved in nervous system development, showed elevated incidence of social anxiety disorder (χ^2^ *p* = 0.03), depression (χ^2^ *p* = 0.049), and Tourette Syndrome (χ^2^ *p* = 0.04) but a decreased incidence of language delay (χ^2^ *p* = 0.01). Together, we find evidence MES risk genes can both contribute to the genetic liability of ASD and its heterogeneity.

While the above results assess phenotypes among single-gene carriers, we also evaluated comorbidities among the subset of individuals with full A*B* MES pair inheritance (*n* = 164 ASD individuals, 135 with comorbidity data). Compared to other ASD cases, carriers of MES gene pairs showed a significantly higher incidence of seizure disorder or epilepsy (χ² *p* = 0.005), and significantly lower rates of ADHD (χ² *p* = 0.004), depression (χ² *p* = 0.002), and oppositional defiant disorder (χ² *p* = 0.04). These findings suggest that full MES pair configurations may shape a distinct neurodevelopmental profile.

### Co-expression and interactions of MES risk gene pairs

Above, we demonstrated that 76 of the 97 predicted MES risk genes exhibited discernible phenotypic associations, further suggesting that MES risk genes do not exclusively form dependent interactions when contributing to ASD risk. To further test dependence, we inquired into the co-expression and protein interaction patterns of these predicted MES risk gene pairs compared to random MES risk gene pairings.

Employing the tissue-based expression data used above, we found that for 48 of 50 gene pairs, the correlation in tissue co-expression did not significantly surpass that observed between any two MES risk genes (Figure S8A). Notably, only the paired genes *CADM3* and *OLIG1* demonstrated a significantly elevated co-expression correlation (*p* = 0.028; Pearson’s *r* = 0.46). However, carriers of *CADM3* demonstrated altered SCQ responses, suggesting the pair’s high co-expression is not compelling evidence of dependence. Interestingly, the other gene pair with significant co-expression, *CACNG8* and *ZNF558*, was anti-correlated (*p* = 0.032; *r* = -0.38). Using the expression profiles of 193 brain subregions, we found three pairs demonstrated significantly elevated co-expression and three pairs with significant anti-correlation (Figure S9A). Together, we do not find evidence that paired MES risk genes are broadly co-expressed.

We additionally clustered MES gene co-expression patterns and compared them to 185 high confidence ASD genes^4^. We found that the predicted MES risk genes did not cluster separately from these established ASD genes, suggesting that MES risk genes integrate into similar tissue-expression pathways (Figure S8B). This same lack of clustering was also observed using the expression from 193 brain subregions (Figure S9B) and fetal brain single-cell RNAseq (Figure S10).

We additionally investigated protein-protein interactions between paired MES risk genes using STRING (v12)^28^. Only one MES pair, *HMGA2* and *CCK*, showed evidence of interaction (score=0.44). Our results do not find strong evidence of co-expression or protein-protein interactions, but given the limitations of STRING and gene expression datasets, this should be interpreted as a lack of supporting evidence for dependent interactions rather than definitive proof of independent contributions.

## Discussion

Within this investigation, we have described 97 predicted MES risk genes in 50 pairs, exploring their connections to brain expression patterns and ASD-like phenotypes. To conclude, we will highlight specific predicted MES risk genes through familial case studies. These cases not only underscore the significance of inheriting multiple MES risk genes but also highlight the disparate contributions of MES risk genes to the genetic susceptibility of ASD.

The concurrent inheritance of ultra-rare deleterious variants in *SNAP25* (Synaptosome Associated Protein 25) and *SLPI* (Secretory Leukocyte Peptidase Inhibitor) emerged as a robust association with ASD (Fig. 1D). *SNAP25*, encoding a pre-synaptic protein, is one of the highest expressed genes in the brain. While variants in *SNAP25* have been linked to ADHD, schizophrenia, bipolar disorder, and neurodevelopmental disorders, it often fails to be linked to ASD^3,4,29,30^. Our findings support this inconsistency as loss-of-function disruptions of one copy of *SNAP25* did not always coincide with ASD, best exemplified by an inherited frameshift variant in a non-ASD mother transmitted to her child with ASD (Fig. 3B). This suggests that meeting the genetic liability for ASD among *SNAP25* carriers necessitates additional contributions from common variations or another MES gene. In the case of the mother-to-son transmitted frameshift in *SNAP25*, the ASD-afflicted child, but not the mother, harbored an ultra-rare deleterious variant in another predicted MES risk gene, *DRGX*, implicated in nervous system development. *SLPI*, another candidate paired with *SNAP25* (Fig. 3B), has recently been implicated in the glia-microbiota-neuronal axis^31^.

Another noteworthy candidate MES risk gene is *DUSP1* (Dual Specificity Phosphatase 1), which encode the a mitogen-activated protein kinase phosphatase (MKP-1) critical for neuronal development^32^. MKP-1 has also been found upregulated in post-mortem brain tissues from individuals diagnosed with major depressive disorder^33^. Our investigation revealed a strong association of *DUSP1* with ASD when co-occurring with *HIST1H4J* (H4 Clustered Histone 11) or *PSMC3* (Proteasome 26S Subunit, ATPase 3), with both genes linked to intellectual disability and neurodevelopmental delay^34,35^. Interestingly, the concurrent inheritance of ultra-rare deleterious variants in *PSMC3* and *HIST1H4J* was observed in only two individuals—a non-ASD mother and her son with ASD (Fig. 3C). Though a limited occurrence, this raises the possibility that *PSMC3* and *HIST1H4J* may have comparatively lesser contributions to the genetic liability of ASD when compared to *DUSP1*. This inference is reinforced by the familial context, as the son with ASD inherited a *DUSP1* variant from the father in addition to the *PSMC3* and *HIST1H4J* variants from the mother. This unique familial pattern suggests a more prominent role for *DUSP1* in the manifestation of ASD within this specific genetic context.

The last candidate MES genes we will illustrate are *SLC8A2* (Solute Carrier Family 8 Member A2) which associates with ASD when paired with either *TERF2* (Telomeric Repeat Binding Factor 2) or *GK5* (Glycerol Kinase 5) (Fig. 3D). *SLC8A2*, a sodium/calcium exchanger, is highly expressed in neurons where it plays a role in sodium and calcium ion homeostasis^36^. *TERF2* plays an important role in telomere maintenance and neuronal differentiation^37^ whereas *GK5* is a broadly expressed gene that is part of glycerol metabolism. The concurrent inheritance of ultra-rare deleterious variants in *TERF2* and *GK5* was observed in four individuals – two with and two without ASD. Three of these individuals were in the same family where variants in both genes were transmitted from a non-ASD father to his two daughters with ASD (Fig. 3D). However, as was observed above with *DUSP1, PSMC3*, and *HIST1H4J*, the daughters with ASD also inherited variants in *SLC8A2* from the mother. This case study again suggests that *SLC8A2* has a greater contribution to the genetic liability of ASD than *TERF2* and *GK5*. In summation, the interplay of our predicted MES genes underscores the polygenic landscape of genetic factors contributing to ASD susceptibility and heterogeneity.

To conclude, this study presents a novel approach to understanding the complex genetic landscape of ASD by identifying MES risk genes – genes that individually, when harboring a deleterious variant, fall below the ASD liability threshold but, when paired with another MES risk gene, collectively contribute to ASD risk. Unlike burden tests, which aggregate rare variants within a single gene, or gene set enrichment analyses, which test predefined pathways, our approach is data-driven and identifies statistical associations between gene pairs without assuming prior biological relationships. This methodology enables the discovery of MES genes that would otherwise remain undetected due to their insufficient effect sizes in isolation.

By analyzing familial inheritance patterns of ultra-rare deleterious variants, we identified candidate gene pairs and further assessed them in 246,825 individuals, including 34,164 with ASD. To ensure these pairwise associations are not driven by stochastic co-inheritance, we required that each pair be observed in at least three ASD-affected families and evaluated their enrichment against expectations derived from gene size and parental variant frequency under an assumption of independent assortment. We ultimately uncovered 97 predicted MES risk genes forming 50 statistically significant gene pairs, where joint inheritance was associated with ASD, despite neither gene showing a significant association individually. These findings underscore the importance of combinatorial genetic contributions to ASD susceptibility and highlight the nuanced interplay of genetic factors influencing ASD risk and heterogeneity. To further contextualize these MES genes, we explored their expression profiles in the brain and examined the phenotypic effects of carriers. Our results are consistent with an additive liability model, wherein MES genes contribute to ASD risk without necessarily acting through monogenic or epistatic mechanisms.

Importantly, this study implemented multiple safeguards against selection bias and winner’s curse, including synonymous variant controls and full-pipeline permutation testing that preserved recurrence filters and familial structures to establish an empirical null distribution. These analyses yielded substantially fewer and weaker associations than observed for deleterious variants. Although only a limited number of pairs were re-observed in SPARK v2 given their extreme rarity, most showed significant enrichment when formally tested, representing a level of independent validation that is uncommon in rare variant studies where replication is often limited by sample size and allele frequency. Nonetheless, confirmation of specific pair conformations will require further investigation, and the present results should be interpreted as statistically supported candidate MES configurations rather than definitive gene-pair validation.

Our study focused on identifying a novel but limited set of paired MES risk genes with the most pronounced associations to ASD. A primary limitation of this study is that we did not consider common genetic variation or environmental conditions, assuming that the co-inheritance of paired MES risk genes alone was sufficient to result in ASD. Further analyses of MES or even large effect size genes may involve comparing carriers with high or low polygenic risk scores, potentially revealing that single MES risk genes and a high burden of common variation may be adequate to cross the diagnostic threshold. The incorporation of common variation may also aid in uncovering even smaller effect size genes. Detecting combinations of three or more genes using our methodology would necessitate a larger number of families. However, identifying smaller effect size genes conditioned on a high or low polygenic risk score remains an avenue for future exploration.

Our investigation sought evidence for whether identified MES risk genes act independently or participate in dependent GxG interactions to influence ASD susceptibility. SCQ responses among non-ASD carriers revealed a significant skew for specific questions, suggesting that many individual MES risk genes may independently contribute to subtle social deficiencies. Furthermore, altered frequencies of developmental or psychiatric conditions in both non-ASD and ASD carriers supported the notion that MES risk genes can independently influence diverse phenotypic outcomes. In total, 76 out of the 97 predicted MES risk genes exhibited skewed frequencies in at least one SCQ response or the incidence of a developmental or psychiatric condition. It is plausible that some MES risk gene pairs form dependent GxG interactions, implying carriers of one gene exhibit no discernable differences from non-carriers. Notably, there were instances where non-ASD individuals harbored ultra-rare deleterious variants in up to five MES risk genes. MES risk gene burden in non-ASD individuals was not associated with sex at birth, suggesting this is not a result of the female protective effect. Instead, in cases where non-ASD individuals possessed more than two MES risk genes, the 21 genes without a skewed frequency in SCQ or developmental/psychiatric conditions constituted an average of 90% of the multiple MES risk genes per individual. If dependent GxG interactions describe this minority of genes, it would allow non-ASD individuals to carry multiple unpaired MES risk genes without affecting their phenotype. This nuanced understanding of the independent and collective actions of MES risk genes provides insights into the intricate nature of their contributions to ASD susceptibility, unraveling the complex interplay between genetics and phenotypic expression.

We hypothesized that MES risk genes would exhibit less conservation than larger effect size genes which was indeed reflected by their moderate pLI/LOEUF deciles. In the context of ASD, genes with large effect sizes typically manifest autosomal-dominant effects, leading to profound phenotypes such as epilepsy, developmental delay, and intellectual disability, alongside ASD^3^. Consequently, de novo mutations in these genes are seldom transmitted to subsequent generations. In contrast, loss-of-function mutations in MES risk genes tend to result in less severe phenotypes if any, possibly due to redundant pathways or functional paralogs. As a result, disruptions in these genes can be transmitted and still contribute to the heritability of ASD. The high heritability of ASD implies that its key genetic factors may generally be subject to more lenient evolutionary constraints. Variants in genes that tolerate altered function and expression may follow similar trends. The ability of MES risk genes to tolerate mutations and still contribute to ASD’s heritability underscores the importance of understanding the interplay between genetic variation, phenotypic outcomes, and evolutionary pressures in the context of ASD susceptibility.

The most challenging aspect of MES risk genes is that the disruption of a single gene is generally inadequate to induce ASD, yet still contributes to its etiology and heterogeneity. This necessitates a nuanced approach in determining how terms such as “causal genes,” “risk genes,” or “pathogenic variants” are to be applied. In the context of monogenic diseases, the causality of a gene or mutation is often straightforward and direct. However, the causality associated with complex diseases like ASD entails a combination of multiple genetic variants and environmental factors contributing to the progression of the condition, rather than merely elevating the risk. Although a MES risk gene may be considered causal as it influences key pathways in ASD development, it does not singularly determine the condition. Guidelines, such as those established by the American College of Medical Genetics and Genomics (ACMG), play a pivotal role in interpreting and categorizing genetic variants, ranging from pathogenic to benign. ACMG incorporates an array of factors, including functional predictions, inheritance patterns, and prior reports, to assign pathogenicity to genes or variants^38^. This approach is highly effective at identifying strong gene drivers of human diseases but less applicable to complex conditions where MES risk genes may play crucial roles. Variants in MES risk genes may be assigned as benign because they are not associated with a disorder, yet carrier status could still predict ASD recurrence or the likelihood of comorbidities. On the other hand, assigning pathogenicity to these variants or genes is also an inaccurate term. This complexity highlights a critical gap in the current genetic interpretation frameworks. As researchers further study complex disorders (e.g., ADHD, OCD, bipolar disorder), there will be a growing need to adapt or develop new guidelines to more accurately address the nuances of polygenic diseases.

## Methods

### Family-based search for MES risk genes in SPARK

Our approach follows a stepwise process with clearly defined statistical thresholds at each stage (Fig. 2). While some empirical filters (e.g., ultra-rare frequency thresholds) are used, all steps are statistically motivated and designed to refine candidates systematically rather than heuristically. All ethical regulations relevant to human research participants were followed. This study uses previously published studies and publicly available datasets and does not involve new human participants.

For our initial family-based screening of MES risk genes, we harnessed a dataset comprising 10,908 families sourced from SPARK^16^ iWES v1 (including sequencing waves WES1-WES4), in which both parents and at least one ASD offspring underwent sequencing via WES. All genotypes were annotated with Ensembl Variant Effect Predictor (VEP)^39^. We excluded genotype calls that did not adhere to Mendelian inheritance patterns within the family (accounting for an average of 0.72% of variants), effectively eliminating de novo mutations. Furthermore, during the initial screening phase, we omitted 113 families in which a proband exhibited a de novo rare deleterious variant (a PTV or a missense variant with a PolyPhen-2^40^ score ≥0.5 or SIFT^41^ score ≤ 0.05; gnomAD v3^42^ non-neuro allele frequency of <1%) in the genes *CHD8, SCN2A, SYNGAP1, SHANK3*, or *ADNP*. These particular genes have a reported zero FDR for association with ASD from TADA^4^, signifying a strong and monogenic-like association with ASD. Consequently, they were deemed unsuitable for the identification of MES risk genes. *PTEN* was not removed for the screen as deleterious variants occurred in controls.

We isolated ultra-rare deleterious variants in the families (a PTV or a missense variant with a PolyPhen-2 score ≥0.5 or SIFT score ≤ 0.05; gnomAD v3 non-neuro allele frequency of <0.1%). Though many more missense pathogenicity estimators are available^43^, we opted for ones that scored for protein function impact without emphasis on species conservation or gene constraint (e.g., MPC^44^). Per our hypothesis, deleterious variants in single MES risk genes do not result in a strong phenotype and may not show as robust evidence of negative selection. For the same reason, we did not exclude genes based on LOEUF or pLI score. Additionally, we only included heterozygous genotypes on the reasoning that (1) homozygous combinations of ultra-rare deleterious variants are effectively unobserved, and (2) individuals homozygous for deleterious variants in a MES risk gene may have a separate non-ASD phenotype.

We first identified familial structures characterized by the inheritance of a specific ultra-rare deleterious variant (denoted as ‘A*‘) by all offspring with ASD within the family, with ‘A*‘ also being present in one non-ASD parent. Subsequently, we identified mirrored patterns of inheritance, focusing on a second variant in a distinct gene (‘B*‘). In such scenarios, ‘B*‘ must derive from the parent who did not transmit ‘A*‘. ‘A*B*‘ must be present in all offspring with ASD and never in non-ASD siblings. Importantly, our methodology accommodated the potential existence of multiple ‘A*‘ or ‘B*‘ variants within the same gene in a single family. We quantified these patterns based on the number of unique families, rather than unique sites. While controlling for the number of transmissions is standard in family-based analyses, our method applies this in a novel way by ensuring that ultra-rare variants in two genes are inherited together only in ASD cases while non-ASD siblings inherit at most one. This allows us to identify MES gene pairs contributing to ASD liability without assuming epistasis.

We next assessed the likelihood of observing ‘B*‘ inheritance patterns within families exhibiting ‘A*‘ inheritance. For this, we conducted binomial samplings to estimate the number of times we should observe any number of secondary independent gene hits:

$$N=\mathop{\sum }\limits_{{gene}=1}^{n{{\rm{\_}}}{genes}}B \sim {Binom}(A,{M}_{{gene}})$$

where B is the number of families with ‘B*’ inheritance, A is the number of families with ‘A*’ inheritance, and M_gene_ is the frequency of ultra-rare deleterious variants in a gene among the 21,570 non-ASD parents. This outputs N, the expected number of genes with B secondary hits given A.

Drawing from the frequency of ultra-rare deleterious variants per gene, calculated using parental data, our aim was to standardize N, representing the expected number of genes with secondary B hits, given A. Notably, larger genes, containing a higher occurrence of ultra-rare deleterious variants, might bear reduced significance when compared to smaller genes with fewer such variants. In our approach, for each pair of candidate MES risk genes, we multiplied N by the relative frequency of ultra-rare deleterious variants in the respective genes, designated as ‘N_norm’. The gene possessing the highest number of ultra-rare deleterious variants, *TTN*, would have an adjustment of one with all other genes adjusted by factors less than one, contingent on their relative frequency of ultra-rare deleterious variants to *TTN*. To illustrate, consider the case of *SNAP25* and *SLPI*. Among the families analyzed, five exhibited the ‘A*‘ inheritance pattern for *SNAP25*, while 21 displayed the same for *SLPI*. Two families featured the mirrored ‘A*B*‘ inheritance pattern for both genes. According to binomial sampling, the occurrence of ‘5-given-2’ is expected approximately 15 times, whereas ‘21-given-2’ should transpire approximately 196 times (before normalization). Due to their smaller gene sizes, *SNAP25* and *SLPI* correspondingly exhibited ‘N_norm’ values of 0.061 and 0.174, indicating that the co-occurrence of these two genes inherited together is relatively unexpected and thus included as a candidate pair for further investigation. We kept pairs where ‘N_norm’ was less than one for both configurations of gene pairs.

### Additional variant types and frequencies

In addition to PTVs and missense variants of ultra-rare or rarer frequency, we tested three other stringencies: PTVs and missense variants of rare (1%) or rarer frequency, only PTVs of ultra-rare (0.1%) and rarer frequency, and only PTVs of rare or rarer frequency. For PTVs and missense variants of rare or rarer frequency, we retained 2507 pairs of genes (2146 total unique genes) where the normalized expected frequency was less than one in both ‘A*‘ and ‘B*‘ configurations. After filtering for non-ASD ‘A*B*’ in the SPARK v1 dataset, only 179 candidate pairs remained (339 total unique genes). For PTVs of rare or rarer frequency, we retained 834 (1336 total unique genes) candidate pairs of which only 83 (161 unique genes) remained after SPARK filtering. For PTVs of ultra-rare or rarer frequency, we retained 692 candidate pairs (1177 total unique genes) of which only 63 (122 unique genes) remained after SPARK filtering.

Of note, the variant frequency thresholds applied in this study do not bias selection toward more or less constrained genes. To demonstrate this, we plotted gene-level LOEUF scores against ultra-rare variant counts and observed that variant burden correlates primarily with coding sequence length rather than constraint (Figure S3C). Accordingly, our filtering strategy relies on functional prediction scores (PolyPhen-2 ≥ 0.5; SIFT ≤ 0.05) without incorporating conservation-based metrics, thereby avoiding systematic enrichment for highly constrained genes.

### Incorporation and assessment of non-neuro cohorts

We included variants from the 3,202 individuals from the 1000 genomes project (1kGP)^17,18^. SIFT scores, PolyPhen-2 scores, and allele frequencies were extracted from GnomAD (v3.1.2)^42^.

Analyses for All of Us were performed in the workspace titled “Detecting the prevalence of ultra-rare gene mutations.” We selected samples with short read whole genome sequencing and excluded individuals with a survey response diagnosis of ASD (diagnosed with, receiving treatment for, or seeking treatment for ASD) or any disorder characterized under the SNOMED term ‘mental disorder. The final cohort included 156,550 individuals of which 50.5% were of a self-reported non-white race and 57.0% were assigned female sex at birth. Due to the security constraints of the All of Us workbench, VEP was performed with SNPeff^45^ (4.3t) as opposed to Ensembl. GnomAD v3 frequencies, Polyphen-2 scores, and SIFT scores were acquired from dbNSFP(v4)^46^. 98.8% of missense variants had an available Polyphen-2 and SIFT score. For high impact insertions and deletions that were not present in dbNSFP, we used the allele frequency in the 156,550 individuals as a proxy. To avoid disseminating individual-level data, we did not report the precise number of individuals with ‘A*B*’ for any gene pair (unless zero or > 20). For any single candidate gene, ultra-rare deleterious variants were always present in at least 20 individuals.

We additionally included individuals from the BioMe Biobank maintained by the Mount Sinai Health System. We isolated 16,586 individuals with genotype information derived from WES and excluded any individuals with a diagnosed or self-reported mental, behavioral, or neurodevelopmental disorders (ICD codes F01-F99). Of the final cohort, the mean age was 61.7 years, 56.2% were of self-reported male sex, and 56.9% were of self-reported non-white race. For consistency, VEP was performed in an identical manner as for the All of Us cohort.

Non-ASD individuals in the SPARK cohort displayed a slightly higher burden of ultra-rare deleterious variants per gene compared to individuals in the other cohorts. On average, candidate genes in the SPARK cohort contained 0.02%, 0.12%, and 0.18% more variants than in the All of Us, BioMe, and 1kGP cohorts, respectively. Across all cohorts, SPARK showed a notable and consistent enrichment for ultra-rare deleterious variants in *UGT2B11*, *AFAP1L2*, *C20orf141*, and *OLAH* (Figure S1). However, these genes also contained relatively more synonymous variants in the SPARK cohort, suggesting their enrichment for deleterious variants is not biologically meaningful. Of note, variants in Z*NF717* were highly enriched in 1kGP compared to the other cohorts (5.74-8.78% more). However, this gene’s role in cellular proliferation and the lymphoblastoid cell line source of all 1kGP samples suggest these variants may be somatic and under positive selection.

To assess potential confounding from population stratification, we analyzed unrelated individuals from 1kGP European (n = 525) and African (n = 686) populations, which represent the predominant ancestries of both ASD cases and controls in this study. For each protein-coding gene, we calculated the collapsed carrier rate of ultra-rare deleterious variants. We then tested for ancestry-associated differences in carrier rates using Fisher’s exact test. Fewer than 1% of genes showed significant differences at p < 0.001, and none of the 97 predicted MES risk genes showed ancestry skew even under Bonferroni correction for 97 tests (p < 0.0005). The only MES gene with nominal association was *RFPL4AL1* (p = 0.0064), which did not pass correction. These results suggest that our gene-level associations are unlikely to be driven by population structure.

### Equilibrium test and Bayes factors

We determined the frequency of ultra-rare deleterious variants per gene across all samples, encompassing both ASD and non-ASD individuals. The probability of ‘A*B*‘ was computed as the product of the individual gene frequencies. Using the R function ‘pbinom,’ we designated this product as the probability of success for ‘A*B*.’ Subsequently, we calculated (1) the probability of observing the given number or a greater number of ASD individuals with ‘A*B*‘, considering the total number of individuals with ASD, and (2) the probability of observing the given number or a lesser number of non-ASD individuals with ‘A*B*‘ mutations, considering the total number of individuals without ASD. The equilibrium probability was then determined as the product of these two probabilities.

For the Bayes factor, the null hypothesis was the equilibrium probability, and the alternate hypothesis was the probability of observing the given number or a greater number of individuals regardless of ASD status with ‘A*B*‘, considering the total number of individuals. The Bayes factor was calculated as the ratio of alternate and null equilibrium probabilities.

### Synonymous variant analyses

We procured ultra-rare synonymous variants across all cohorts, excluding those with a secondary functional impact (e.g., missense, or more significant effects) on another gene. Ultra-rare synonymous variants were, on average, 1.83 times more frequent per gene than ultra-rare deleterious variants, which underwent further filtration for deleteriousness via VEP. We determined the ratio of ultra-rare synonymous to ultra-rare deleterious variants across all protein-coding genes. As some genes presented markedly divergent ratios, we excluded those beyond the >95th or <5th percentile thresholds. This adjustment predominantly impacted smaller genes where variants were only observed in a few samples.

Using ultra-rare synonymous variants, the candidate screening (Fig. 2) identified 690 candidate gene pairs, significantly fewer than the 1916 pairs identified with deleterious variants. Subsequent analysis incorporating the complete SPARK v1 cohort (Fig. 2) further narrowed the candidate pairs to 129 (258 unique genes).

In the SPARK v1 cohort, we noted outlier individuals with high counts of ultra-rare synonymous variants. On average, the occurrence of ultra-rare synonymous variants was less than one per individual per gene. Among 258 unique genes from 129 candidate pairs identified with synonymous variants, four samples exhibited over 100 variants in single genes, 50-fold higher than the mean (SP0122762, SP0379928, SP0149880, SP0064380; Figure S3D). The excess synonymous variants in the four samples were unique and not cataloged in gnomAD v4, casting doubts on their quality. Contrastingly, such an excess was not observed with ultra-rare deleterious variants (Figure S3D). We subsequently removed these four samples from analyses of synonymous variants.

### Incorporation of SPARK v2 and capacity for rare variant replication

For additional validation, we incorporated non-overlapping samples from SPARK iWES v2 (WES5). Quality control was identical to that of SPARK v1. To calculate the expected number of replicated pairs in SPARK v2, we conducted simulations based on the frequency of A*B* events in ASD from SPARK v1. These simulations demonstrated that, among 10,128 ASD cases (the number in SPARK v2), we can expect to observe A*B* at least once for an average of 8.6 pairs with a standard deviation of 2.7 pairs. This finding indicates that the eight pairs we observed in this independent dataset are entirely within expected ranges. Broad replication would likely require much larger datasets, with our simulations suggesting that cohorts of approximately 150,000 ASD cases would be necessary to robustly replicate even half of the observed pairs.

### Simulation of discovery pipeline

To create a null distribution for the number and significance of MES gene pairs, we simulated carrier statuses for all 246,825 individuals in the main cohorts, preserving Mendelian inheritance patterns in families, and performed the MES discovery pipeline in 1000 iterations. For simulating variant assignments, we first simulated carrier statuses for “orphans”, defined as all individuals without either parent in any cohort. We used the R function ‘rbinom’ to sample from all protein coding genes using the per-gene ultra-rare deleterious variant carrier probabilities. From the orphans, we next simulated the carrier statuses of any offspring, which were all within the 1kGP and SPARK cohorts. For these offspring with parents represented in the cohorts, we directly used the carrier statuses of the parents, allowing each variant a 50% probability of transmission using ‘rbinom’. If only one parent was represented, which occurs frequently in SPARK, we simulated the missing parent’s carrier statuses and performed the same transmission test. This creates more realistic carrier statuses among siblings where only one parent is available in the cohort. This step does not include simulating de novo variants as these are pruned in the real analyses. For identical twins, the carrier statuses were corrected to match by randomly selecting one twin to represent both. In each iteration, we performed the MES discovery pipeline identically (Fig. 2). We compared the non-normal distributions of real versus simulated equilibrium probabilities using the Wilcoxon rank sum test (R command ‘wilcox.test’).

### Screening for high-effect variant confounds

While we the discovery screen excluded individuals with de novo variants in consensus high-penetrance ASD genes, we did not exclude all possible pathogenic variants, as many loci lack consensus on penetrance or effect size. To assess potential confounding, we reviewed CLIA-confirmed clinical results (~2.16% of ASD cases) and identified four individuals affecting three MES pairs: *C2orf50/CAMK2B* (n = 2), *CBLN4/CORO6*, and *OLAH/KLHL42* (Data S6). We now flag the first and third as potential false positives. In the remaining case, a *de novo EHMT1* variant (missense, ClinVar variant of unknown significance) co-occurred with the MES pair only in one of two affected siblings in a multiplex family, and did not segregate with ASD, supporting the relevance of the MES configuration. Our exclusions aim to remove variants with clear monogenic effects while preserving moderate inherited risk consistent with our model.

### Gene expression

We used nTPM values derived from RNAseq from the Human Protein Atlas (v23)^21^. The 50 tissue consensus used transcriptomics from both the Human Protein Atlas and the GTEx consortium^47^. Expression for the 193 brain subregions was also from the Human Protein Atlas. To test if the 97 predicted MES risk genes were significantly enriched in a tissue or brain subregion, we sampled genes from all protein coding genes without replacement in 1,000 iterations. For each iteration, we measured the fraction of sampled genes expressed (nTPM >1) in each tissue or brain subregion. We used the Shapiro–Wilk test^48^ (R command ‘shapiro.test’) to confirm sampled statistics were normally distributed (p > 0.05). We then used the cumulative density function of the normal distribution to determine the significance of predicted MES risk gene enrichment per tissue or brain subregion.

Using the same two RNAseq datasets described above along with single-cell RNAseq from first trimester fetal brain^49^, we calculated the correlation of nTMP values across tissues and brain subregions. To assess the significance of MES pair co-expression, we calculated the co-expression correlation of all possible MES risk gene pairs and then calculated the p-value of real MES pairs using the R function ‘pnorm’. Hierarchical clustering of the co-expression of MES and other ASD genes was performed with the R function ‘hclust’ using the ‘ward.D2’ method and plotted with ‘ComplexHeatmap’^50^.

### Phenotypes of MES risk gene carriers

We utilized the Social Communication Questionnaire (SCQ) and basic medical questionnaire administered in the SPARK v1 cohort. For the SCQ total score, we removed 167 non-ASD siblings with a score at or above 13 (two standard deviations above the mean among non-ASD respondents). For the basic medical screening, we excluded questions related to birth defects (as they were uncommon) and environmental exposures (e.g., lead poisoning, fetal alcohol syndrome, traumatic brain injury).

To assess the significance of the number of skewed results for both SCQ questions and neurodevelopmental disorder incidence, we randomly shuffled the identity of carriers, preserving the number of carriers in 1,000 iterations. P-values were calculated with the R command ‘pnorm’ (shuffling statistics were normally distributed via Shapiro–Wilk test; two-sided). We did not evaluate the number of significant questions across all MES risk genes, as the variable number of carriers per gene produces non-comparable tests.

### Reporting summary

Further information on research design is available in the Nature Portfolio Reporting Summary linked to this article.

## Supplementary information

Supplementary Information

Description of Additional Supplementary Files

Supplementary Data S1-S7

Reporting Summary

## Data Availability

Whole genome sequencing from 1kGP is freely available from The International Genome Sample Resource (https://www.internationalgenome.org/). Allother individual-level data used in this study are not publicly available with access permission subject to review. The genetic and phenotypic data for SPARK can be requested at SFARI Base (https://www.sfari.org/resource/spark/). For convenience, we have included all ultra-rare deleterious variants in the SPARK cohort (Data S7). Access to the Sinai BioMe data can be requested through The Charles Bronfman Institute for Personalized Medicine (https://icahn.mssm.edu/research/ipm/programs/biome-biobank) and is subject to compliance with the Mount Sinai Health System’s data use agreements. All of Us controlled tier data is available for registered institutions and cohort selection is detailed in the workbench titled “Detecting the prevalence of ultra-rare gene mutations.”

## Code availability

Code and resources used in this study is available both on GitHub at (https://github.com/MahjaniLab/MES_Code) and Zenodo (10.5281/zenodo.19859234). For any further inquiries or requests for code not available in the repository, please contact the corresponding author.
