# Supplementary material for: Identification of moderate effect size genes in autism spectrum disorder through a novel gene pairing approach
Source: Commun Biol. 2026 May 30;9:1146. doi: 10.1038/s42003-026-10380-z (PMC13518923; doi:10.1038/s42003-026-10380-z)
Supplement: Supplementary file 1 [file 42003_2026_10380_MOESM1_ESM.pdf]

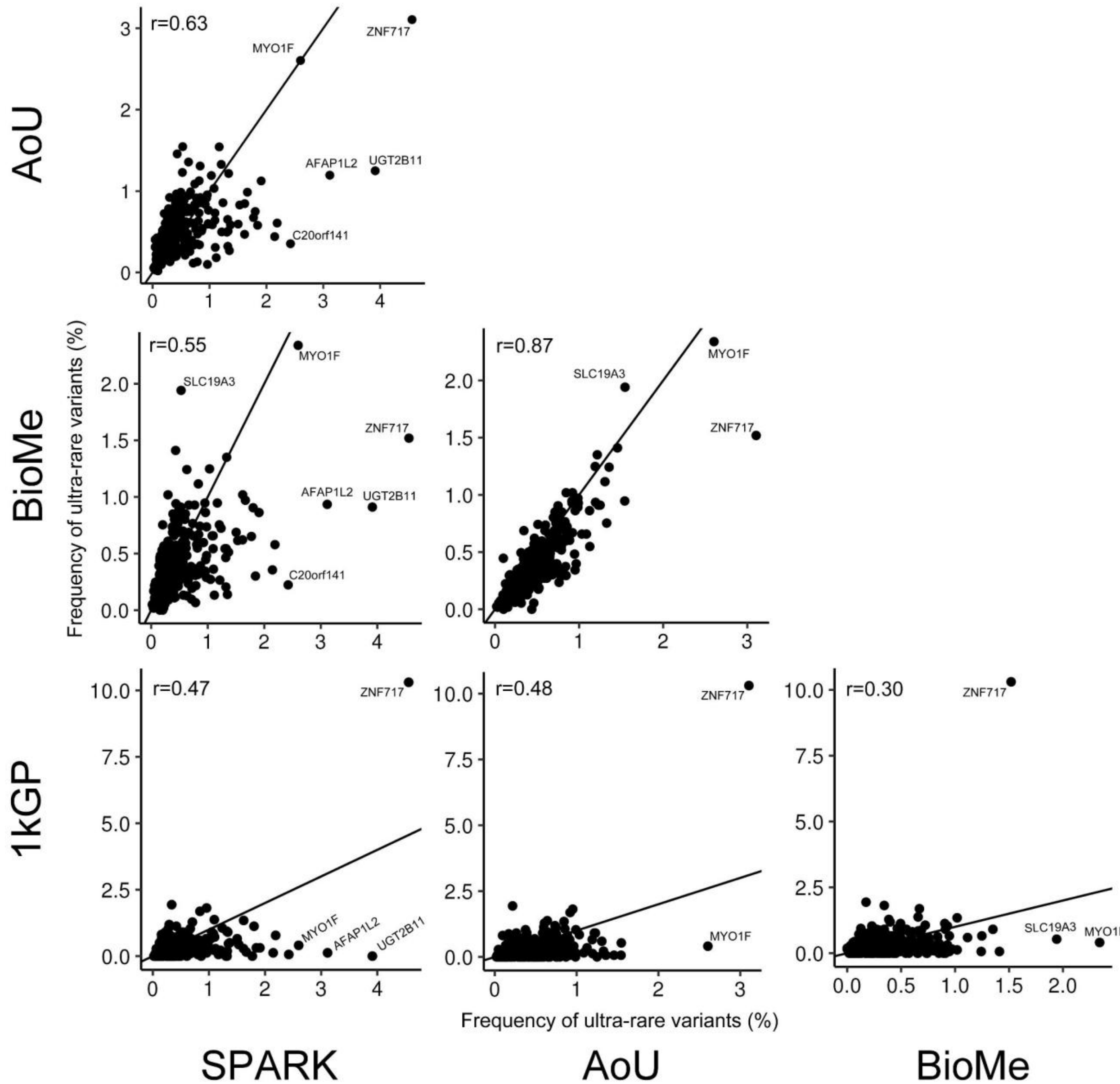

Figure S1. Comparison of ultra-rare variant frequency by MES candidate gene across cohorts. The percent of individuals per cohort with at least one ultra-rare deleterious variant in 318 candidate MES genes. The line represents linear regression. Pearson correlation coefficients are included for each plot.

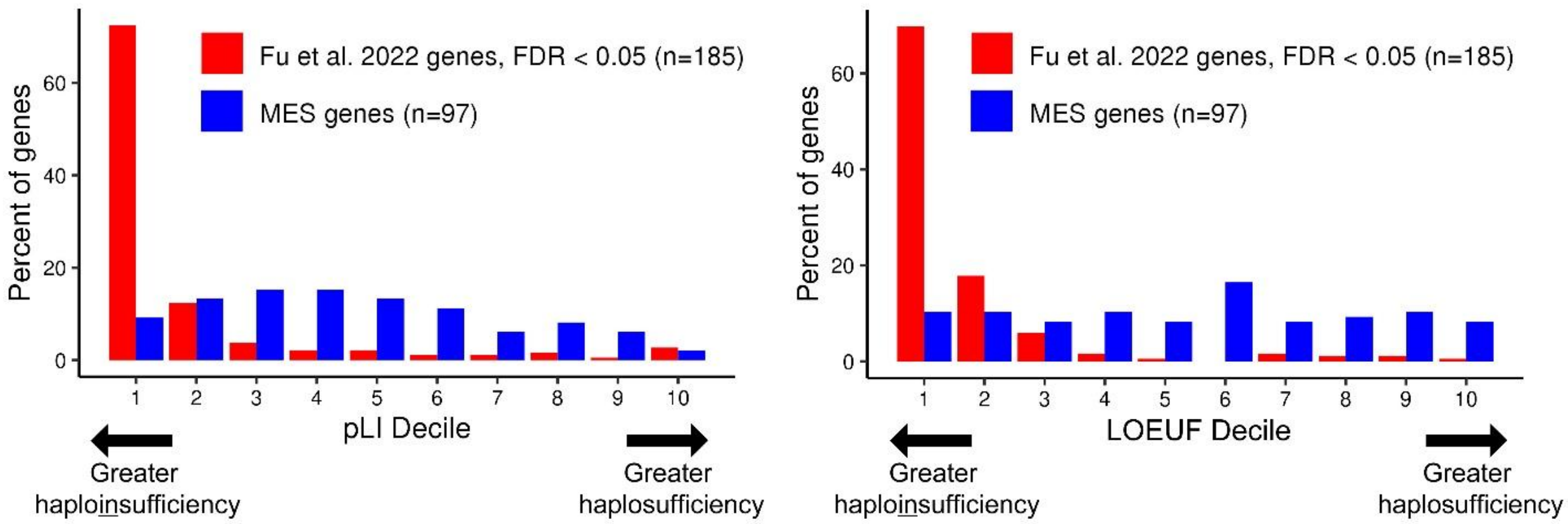

Figure S2. Distribution of pLI and LOEUF scores for ASD genes. Representation of MES genes and genes described in Fu et al. 2022 in decile bins created from the probability of loss of function intolerance (pLI) and loss-of-function observed over expected upper bound fraction (LOEUF) score of all protein coding genes.

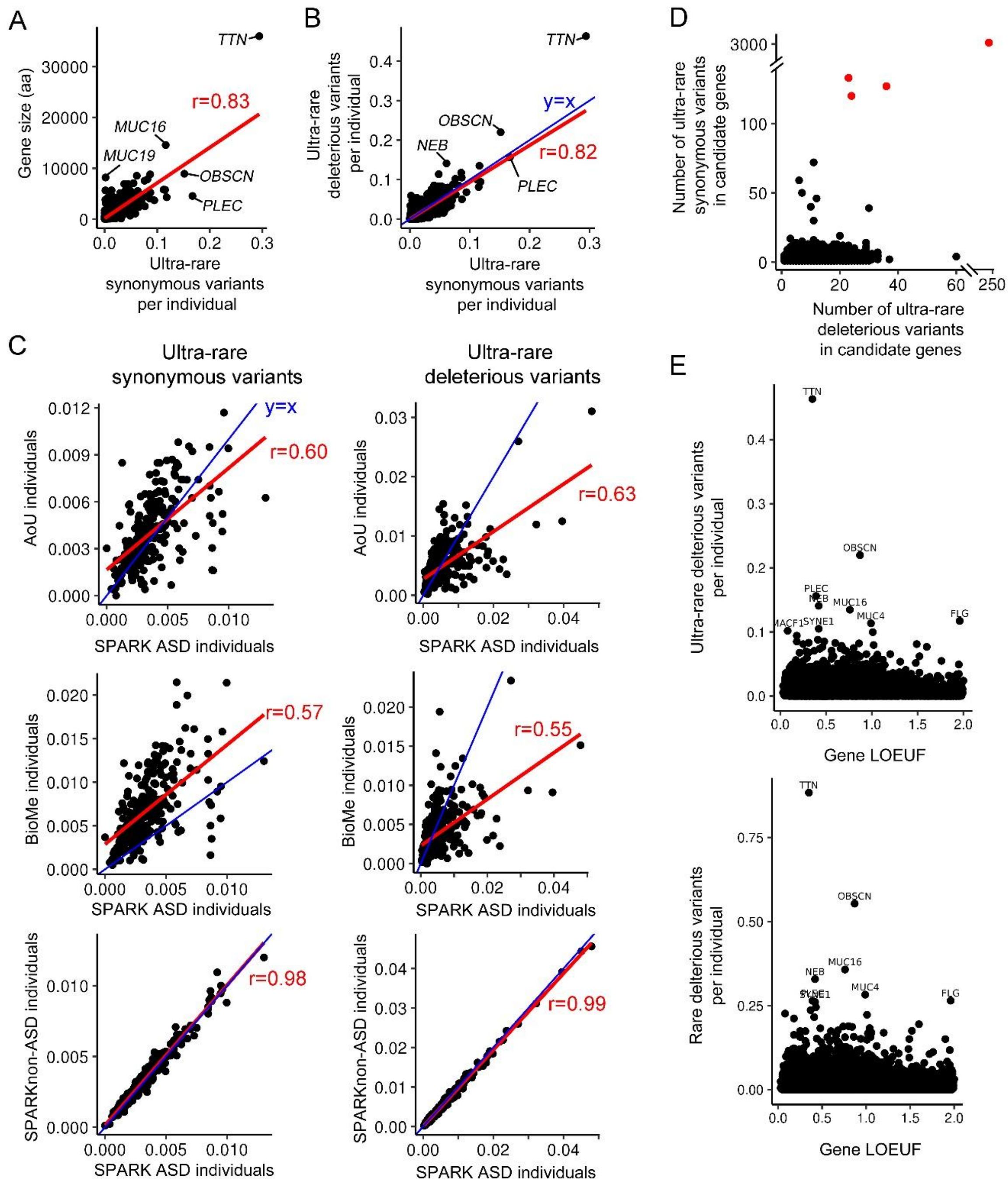

Figure S3. Validation with ultra-rare synonymous variants. (A) Mean number of ultra-rare (<0.1%) synonymous variants in SPARK parents versus gene size. The red line represents linear regression. (B) Mean number of ultra-rare synonymous variants in SPARK v1 parents versus the mean number of ultra-rare deleterious variants (missense and PTVs) as shown in Fig 1C. (C) Comparison of ultra-rare synonymous variant frequencies in candidate genes across controls cohorts to ASD individuals in SPARK v1. Of note, the candidate genes discovered with synonymous variants differed from those with deleterious variants. (D) The number of ultra-rare synonymous and deleterious variants in candidate genes among the SPARK v1 cohort. The four individuals highlighted in red were removed from analyses. (E) As in Fig 1C, comparing to gene LOEUF score.

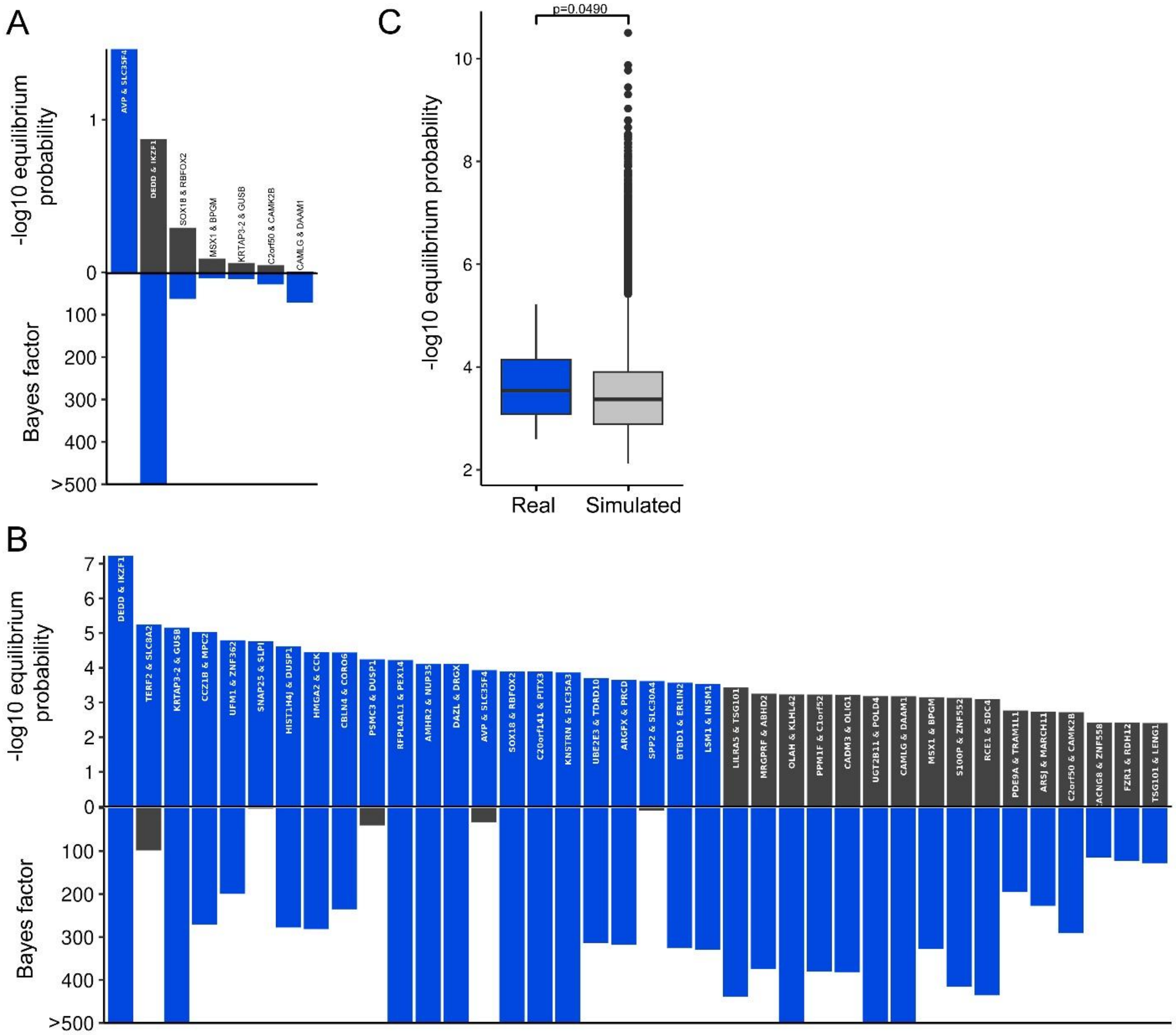

Figure S4. Validation of MES genes with SPARK v2. (A) As in Fig 1D, equilibrium probability and Bayes factors for the predicted MES gene pairs in ASD using SPARK v2 in place of SPARK v1. Bars in blue denote an equilibrium probability <0.05 or a Bayes factor >10. Only seven of the eight pairs with at least one A*B* in ASD are tested. (B) As in Fig 1D combining SPARK v1 and v2 samples. (C) Equilibrium probabilities for the 50 real MES gene pairs and those from 1,000 simulations. P-value is calculated from a one-sided Wilcoxon signed-rank test.

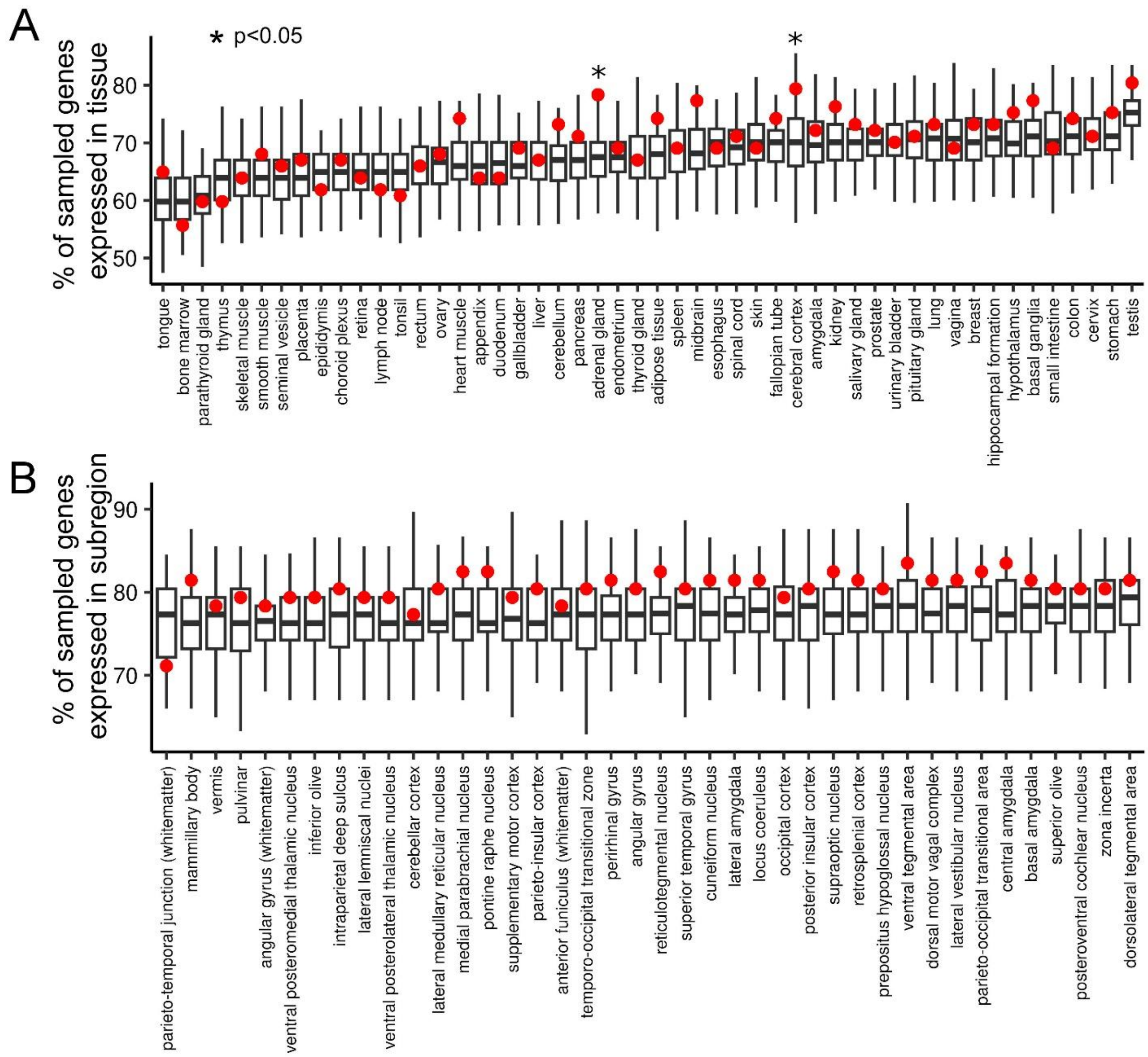

Figure S5. Fraction of MES genes expressed per tissue compared to simulations. (A) Red point: The actual proportion of the 97 predicted MES genes expressed (nTPM > 1) in the corresponding tissue. Boxplots: The proportions of sampled protein-coding genes expressed in the respective tissue over 1000 iterations. Outliers are removed. Significant tissues are highlighted. (B) As in panel A for brain subregions. Only a subset of the 196 subregions is displayed.

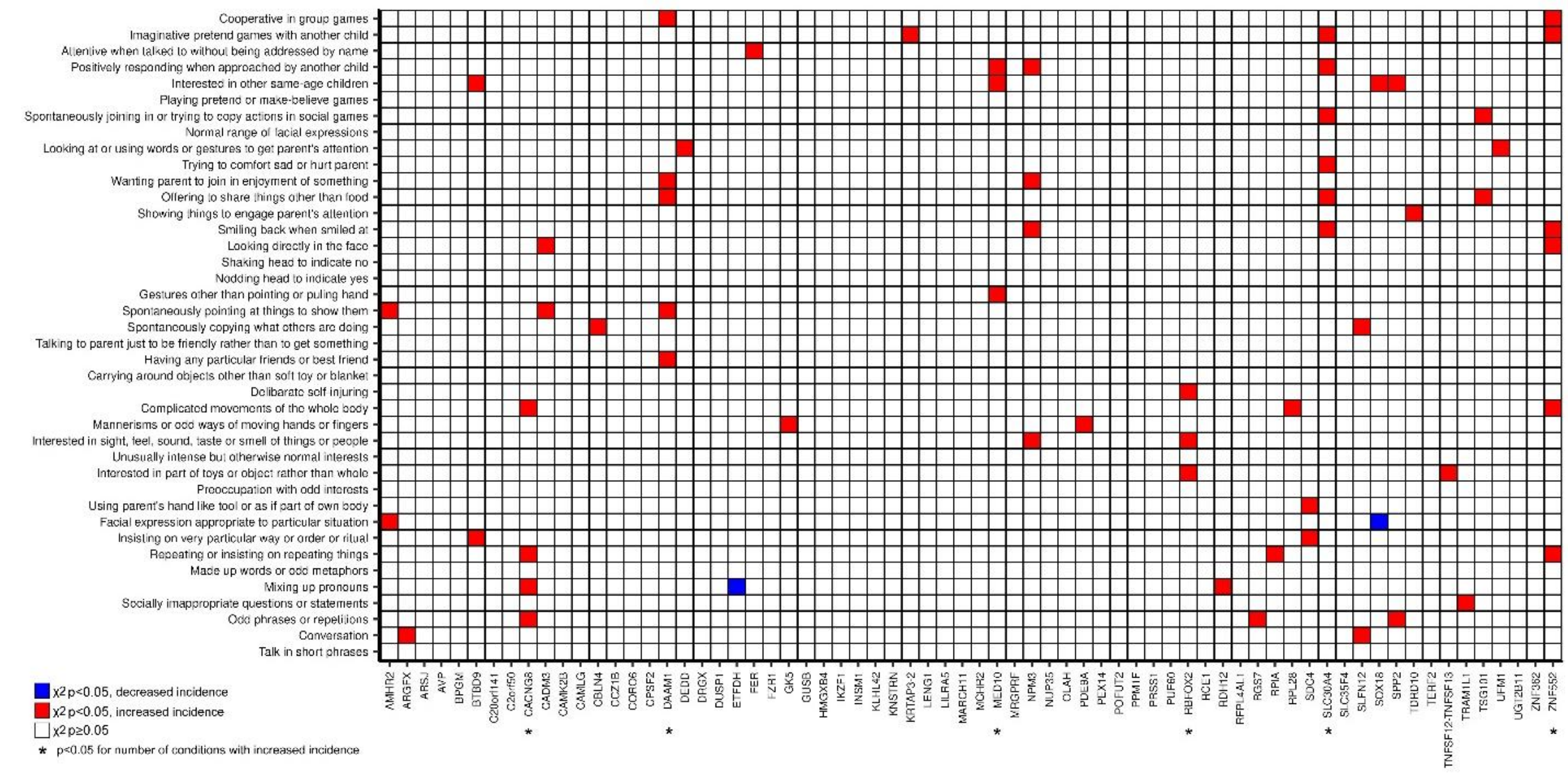

Figure S6. SCQ response skew in non-ASD carriers of MES genes. Shown are the 68 MES genes with at least five non-ASD gene carriers with SCQ responses. Genes with a χ2 $p<0.05$ differences in carrier versus non-carrier responses are highlighted. In 63 of the 65 significant questions, carriers showed greater ASD-like responses, colored red. The remaining two showed fewer ASD-like responses, colored in blue. Genes where the number of skewed responses is significant ($p<0.05$) compared to shufflings are noted with an asterisk.

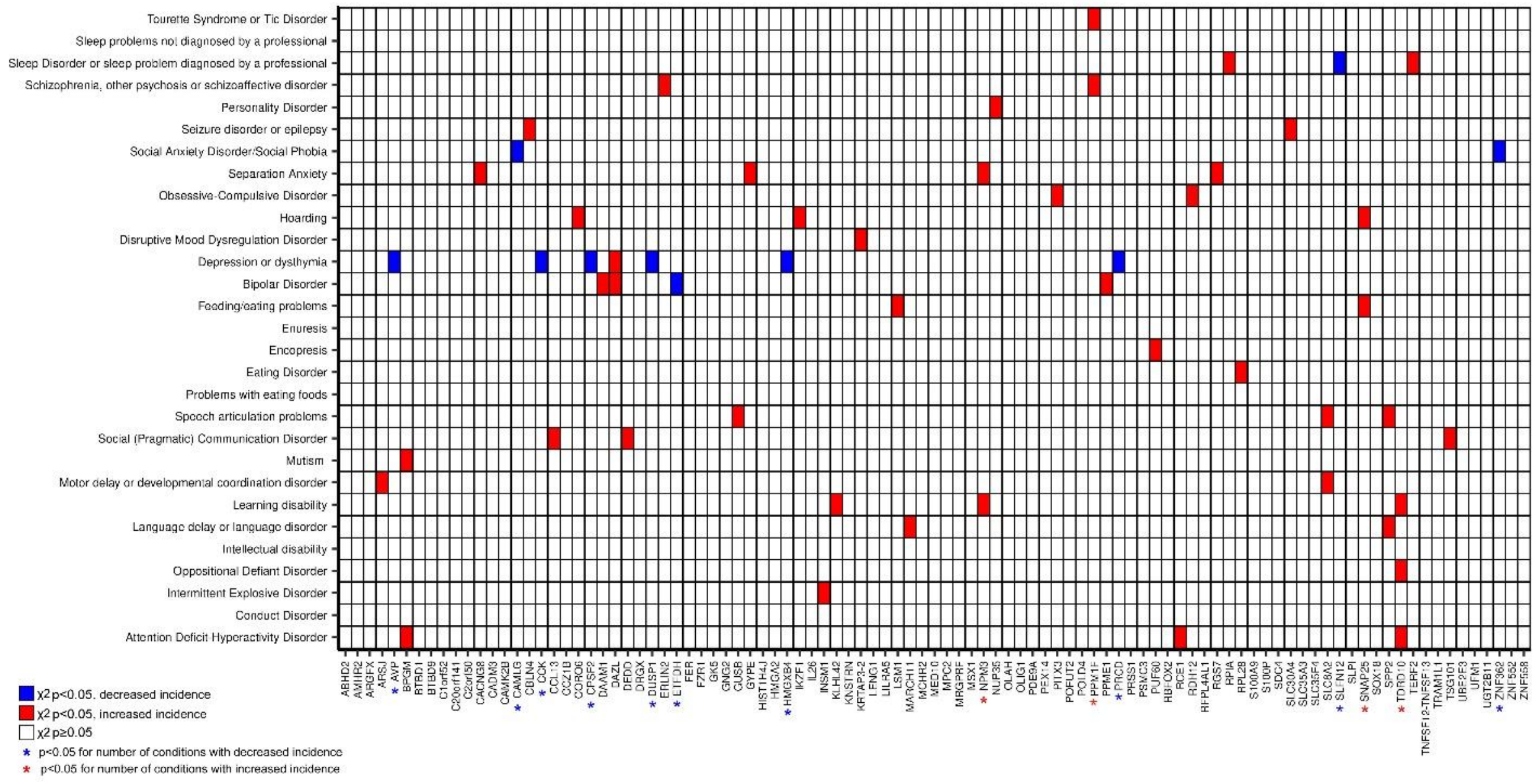

Figure S7. Neurodevelopmental condition skew in non-ASD carriers of MES genes. Genes with a χ2 p<0.05 difference in carrier versus non-carrier condition frequencies are highlighted. In 51 of the 61 highlighted cases, carriers showed greater condition frequency, colored red. The remaining ten show decreased condition frequency, colored blue. Genes where the number of skewed responses increasing or decreasing incidence is significant (p<0.05) compared to shufflings are noted with an asterisk.

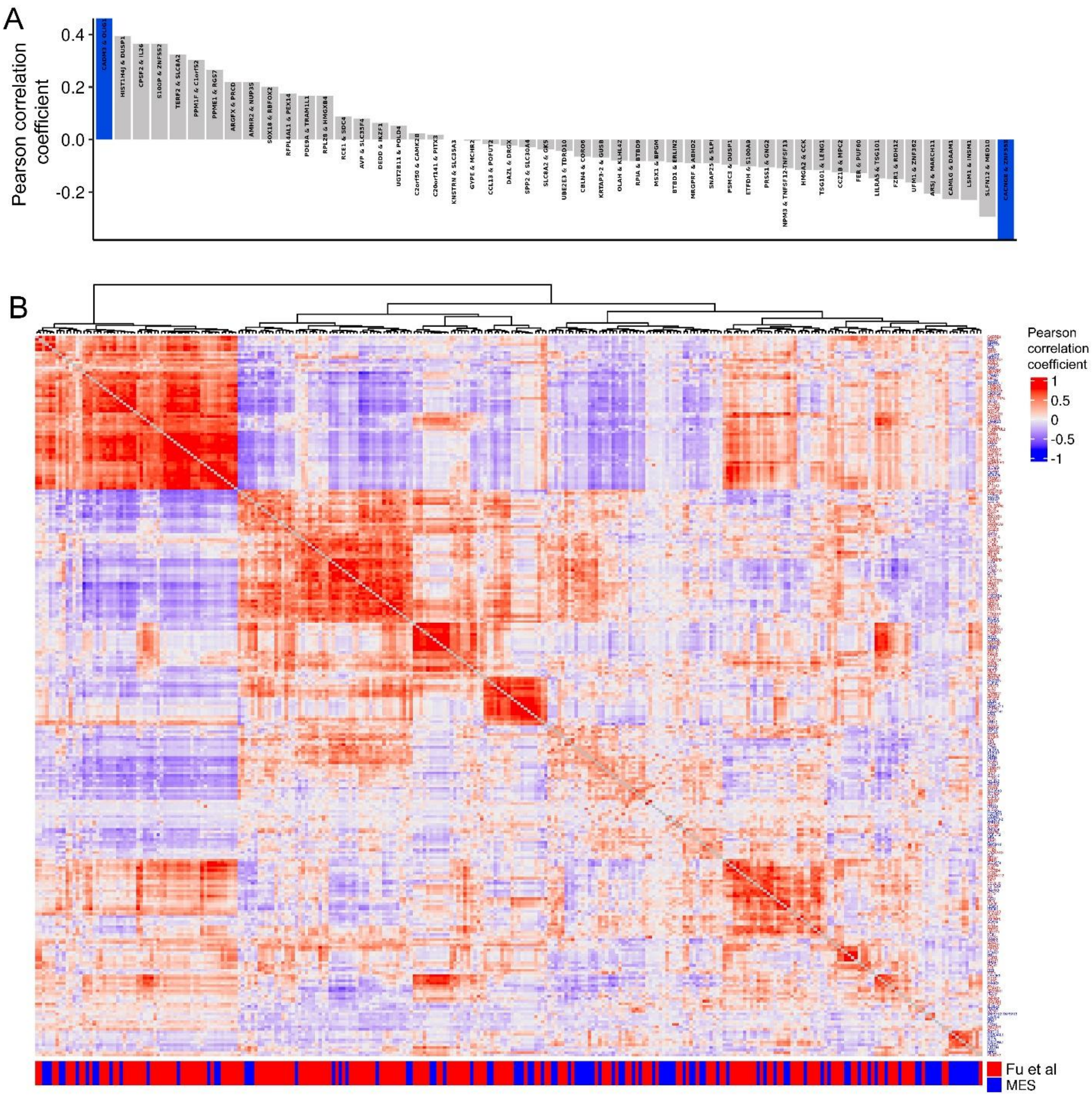

Figure S8. Co-expression of ASD genes across 50 tissues. (A) The correlation of nTMP values of paired MES genes. Bars highlighted in blue indicate significantly higher or lower correlation ($p<0.05$) compared to any two MES genes. (B) Clustered co-expression of the 97 MES genes (labeled in blue) and 185 genes ASD-associated genes described in Fu et al. 2022 (FDR < 0.05) (labeled in red).

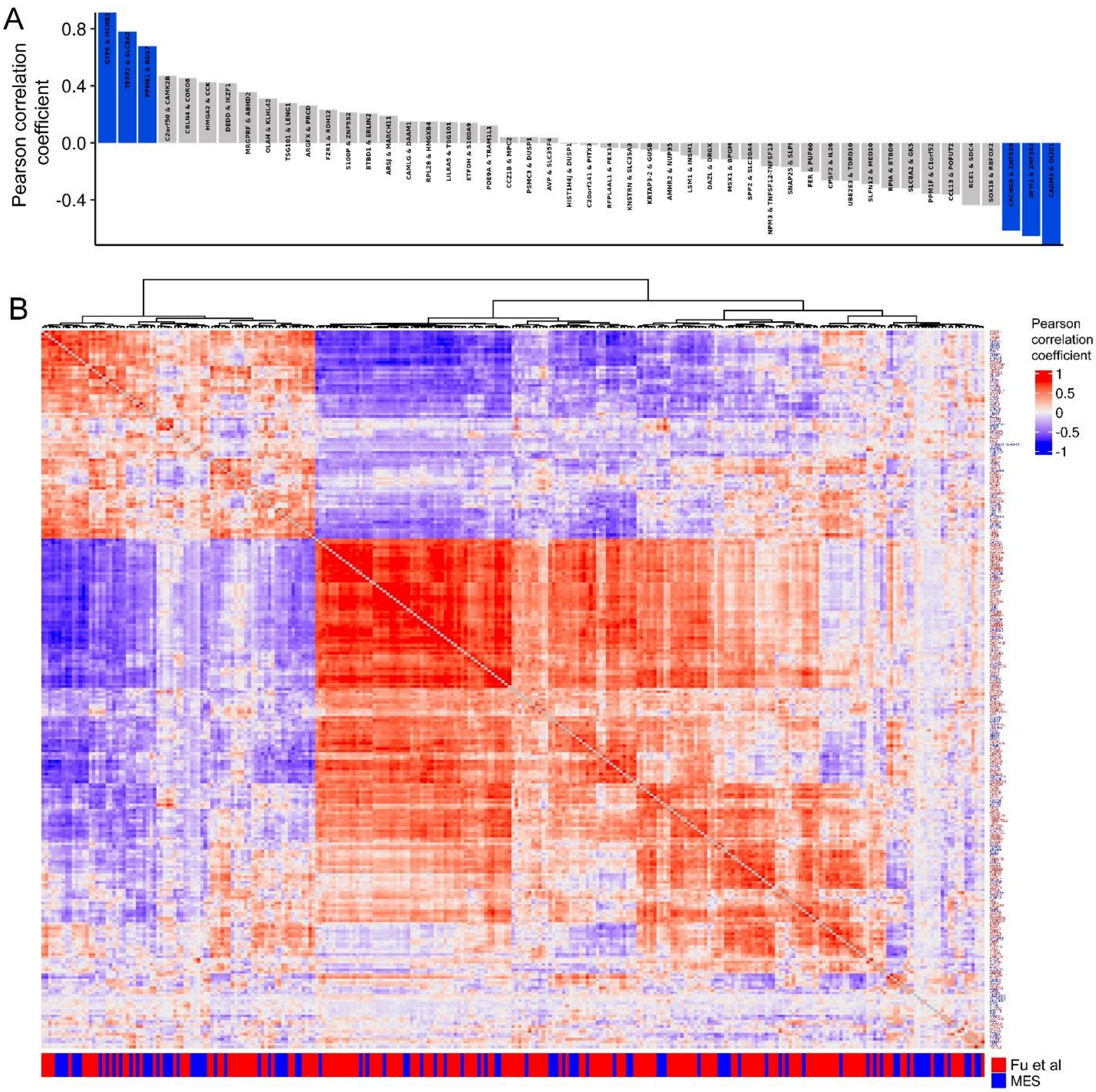

Figure S9. Co-expression of ASD genes across 193 brain subregions. (A) As in Fig S8, the correlation of nTMP values of paired MES genes. Bars highlighted in blue indicate significantly higher or lower correlation ($p<0.05$) compared to any two MES genes. Two pairs (*PRSS1/GNG2* and *UGT2B11/POLD4*) could not be tested due to undefined subregion expression. (B) As in Fig S8, clustered co-expression of the 97 MES genes (labeled in blue) and 185 genes ASD-associated genes described in Fu et al. 2022 (FDR < 0.05) (labeled in red).

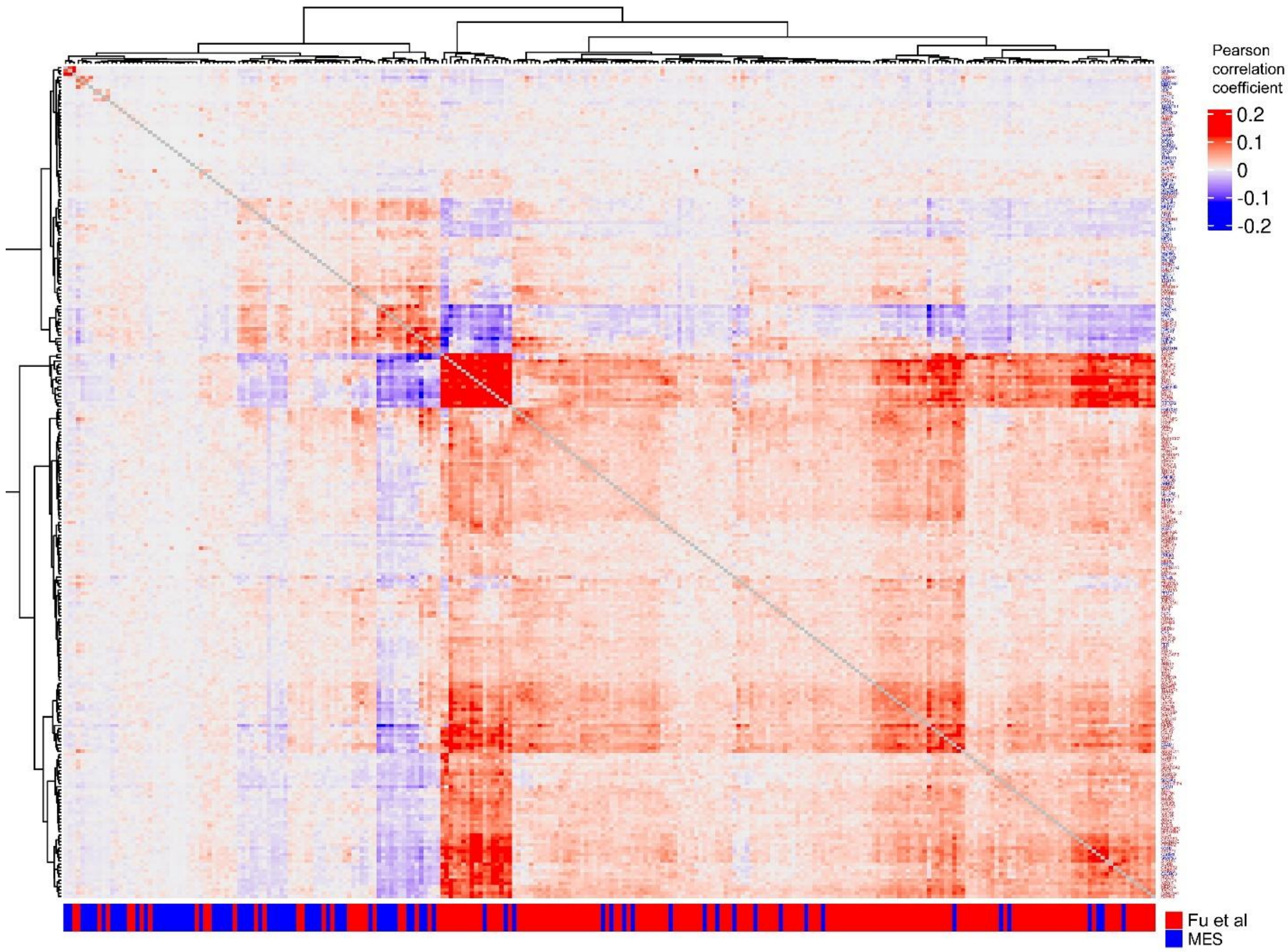

Figure S10. Co-expression of ASD genes in fetal brain single-cell RNAseq. Clustered co-expression of the 97 MES genes (labeled in blue) and 185 genes ASD-associated genes described in Fu et al. 2022 (FDR < 0.05) (labeled in red).
