# Supplementary material for: Identification of moderate effect size genes in autism spectrum disorder through a novel gene pairing approach
Source: Commun Biol. 2026 May 30;9:1146. doi: 10.1038/s42003-026-10380-z (PMC13518923; doi:10.1038/s42003-026-10380-z)
Supplement: Supplementary file 2 [file 42003_2026_10380_MOESM2_ESM.pdf]

Description of Additional Supplementary Files

File name: Supplementary Data
Description: Supplemental data tables S1-S7. Legends are included.
